# Risk factors for SARS-CoV-2 infection after primary vaccination with ChAdOx1 nCoV-19 or BNT162b2 and after booster vaccination with BNT162b2 or mRNA-1273: A population-based cohort study (COVIDENCE UK)

**DOI:** 10.1016/j.lanepe.2022.100501

**Published:** 2022-09-23

**Authors:** Giulia Vivaldi, David A. Jolliffe, Hayley Holt, Florence Tydeman, Mohammad Talaei, Gwyneth A. Davies, Ronan A. Lyons, Christopher J. Griffiths, Frank Kee, Aziz Sheikh, Seif O. Shaheen, Adrian R. Martineau

**Affiliations:** a Blizard Institute, Barts and The London School of Medicine and Dentistry, Queen Mary University of London, London, UK; b Wolfson Institute of Population Health, Barts and The London School of Medicine and Dentistry, Queen Mary University of London, London, UK; c Asthma UK Centre for Applied Research, Queen Mary University of London, London, UK; d Population Data Science, Swansea University Medical School, Singleton Park, Swansea, UK; e Centre for Public Health Research (NI), Queen's University Belfast, Belfast, UK; f Usher Institute, University of Edinburgh, Edinburgh, UK

**Keywords:** SARS-CoV-2, Vaccination, Breakthrough infection, ChAdOx1, BNT162b2, mRNA-1273

## Abstract

**Background:**

Little is known about how demographic, behavioural, and vaccine-related factors affect risk of post-vaccination SARS-CoV-2 infection. We aimed to identify risk factors for SARS-CoV-2 infection after primary and booster vaccinations.

**Methods:**

This prospective, population-based, UK study in adults (≥16 years) vaccinated against SARS-CoV-2 assessed risk of breakthrough SARS-CoV-2 infection up to February, 2022, for participants who completed a primary vaccination course (ChAdOx1 nCoV-19 or BNT162b2) and those who received a booster dose (BNT162b2 or mRNA-1273). Cox regression models explored associations between sociodemographic, behavioural, clinical, pharmacological, and nutritional factors and test-positive breakthrough infection, adjusted for local weekly SARS-CoV-2 incidence.

**Findings:**

1051 (7·1%) of 14 713 post-primary participants and 1009 (9·5%) of 10 665 post-booster participants reported breakthrough infection, over a median follow-up of 203 days (IQR 195–216) and 85 days (66–103), respectively. Primary vaccination with ChAdOx1 (*vs* BNT162b2) was associated with higher risk of infection in both post-primary analysis (adjusted hazard ratio 1·63, 95% CI 1·41–1·88) and after an mRNA-1273 booster (1·26 [1·00–1·57] *vs* BNT162b2 primary and booster). Lower risk of infection was associated with older age (post-primary: 0·97 [0·96–0·97] per year; post-booster: 0·97 [0·97–0·98]), whereas higher risk of infection was associated with lower educational attainment (post-primary: 1·78 [1·44–2·20] for primary/secondary *vs* postgraduate; post-booster: 1·46 [1·16–1·83]) and at least three weekly visits to indoor public places (post-primary: 1·36 [1·13–1·63] *vs* none; post-booster: 1·29 [1·07–1·56]).

**Interpretation:**

Vaccine type, socioeconomic status, age, and behaviours affect risk of breakthrough infection after primary and booster vaccinations.

**Funding:**

Barts Charity, UK Research and Innovation Industrial Strategy Challenge Fund.

**Research in context**

**Evidence before this study**

We searched PubMed, medRxiv, and Google Scholar for papers published up to Feb 18, 2022, using the search terms (breakthrough OR post-vaccin*) AND (SARS-CoV-2 OR COVID) AND (disease OR infection) AND (determinant OR “risk factor” OR associat*), with no language restrictions. Existing studies on risk factors for breakthrough SARS-CoV-2 infection among vaccinated individuals have found associations with age, comorbidities, vaccine type, and previous infection; however, findings have been inconsistent across studies. Most studies have been limited to specific subgroups or have focused on severe outcomes, and very few have considered breakthrough infections after a booster dose or have adjusted for behaviours affecting exposure to other people.

**Added value of this study**

This study is among the first to provide a detailed analysis of a wide range of risk factors for breakthrough SARS-CoV-2 infection, both after the primary course of vaccination and after a booster dose. Our large study size and granular data have allowed us to investigate associations with various sociodemographic, clinical, pharmacological, and nutritional factors. Monthly follow-up data have additionally given us the opportunity to consider the effects of behaviours that may have changed across the pandemic, while adjusting for local SARS-CoV-2 incidence.

**Implications of all the available evidence**

Our findings add to growing evidence that risk factors for SARS-CoV-2 infection after primary or booster vaccinations can differ to those in unvaccinated populations, with effects attenuated for previously observed risk factors such as body-mass index and Asian ethnicity. The clear difference we observed between the efficacies of ChAdOx1 nCoV-19 and BNT162b2 as the primary course of vaccination appears to have been reduced by the use of BNT162b2 and mNRA-1273 boosters. As more countries introduce booster vaccinations, population-based studies with longer follow-up will be needed to investigate our findings further.

Alt-text: Unlabelled box

## Introduction

Vaccination against SARS-CoV-2 has been a key strategy to control the COVID-19 pandemic. With more than 12·57 billion doses administered worldwide, and more than 67% of the world's population having received at least one dose by August, 2022,^1^ we are moving into the post-vaccination era. Vaccination substantially reduces both COVID-19 disease severity and mortality,^2^ but its effects on transmission appear to be more modest.^3^ Additionally, waning protection^4^ and the emergence of variants with increased transmissibility and immune evasion^5^ increase the likelihood of post-vaccination, or breakthrough, infections.

The picture is further complicated by the introduction of so-called booster doses of SARS-CoV-2 vaccines. The emergence of the Omicron variant of concern led to the expedited booster rollout in the UK,^6^ where more than 65% of the population aged 12 years and older had received a booster or third vaccine dose by March 1, 2022.^7^ The vaccinated population in the UK remains split into two main groups: those who have received a complete primary course and those who have additionally received an (often heterologous) booster dose. As more countries roll out booster programmes,^8^ both groups need to be studied to fully capture their respective risks for breakthrough SARS-CoV-2 infection, and to better understand the effects of booster vaccinations.

While much research has been done on risk factors for SARS-CoV-2 infection in unvaccinated populations, risk factors for breakthrough infection in vaccinated individuals are less well understood and existing studies present conflicting findings. Whereas some studies have reported a higher risk of breakthrough infection among individuals with comorbidities,^9^^,^^10^ other large-scale studies have found no associations with pre-existing conditions.^2^ Similarly, several studies have suggested that older age is associated with increased risk of breakthrough infections,^11^^,^^12^ whereas others have found no association^13^^,^^14^ or an inverse association.^2^ Most studies on risk of breakthrough infection have been limited to specific subgroups,^10^^,^^13^^,^^15^ focused solely on severe outcomes,^16^^,^^17^ or only considered infections before a booster dose.^2^^,^^9^ Additionally, the few studies considering behaviours, such as levels of physical activity or journeys on public transport, have relied on baseline values,^2^ and so have been unable to capture changes in behaviour that may have occurred over the various stages of the pandemic.

We therefore did a prospective, nationwide, population-based study in UK adults to investigate and compare the risk factors for breakthrough SARS-CoV-2 infection after primary vaccination and after booster vaccination. We included data on three widely used vaccines and considered a range of potential sociodemographic, clinical, pharmacological, and nutritional determinants of response to vaccination and susceptibility to infection, as well as behavioural factors derived from monthly follow-up questionnaires.

## Methods

### Study design and participants

COVIDENCE UK is a prospective, longitudinal, population-based observational study of COVID-19 in the UK population (https://www.qmul.ac.uk/covidence). Inclusion criteria were age 16 years or older and UK residence at enrolment, with no exclusion criteria. Participants were invited via a national media campaign to complete an online baseline questionnaire and monthly follow-up questionnaires to capture information on potential symptoms of COVID-19, results of nose or throat swab tests for SARS-CoV-2, COVID-19 vaccination status, and details of a wide range of potential determinants of vaccine response and SARS-CoV-2 exposure. The study was launched on May 1, 2020, and closed to enrolment on Oct 6, 2021. Further details on COVIDENCE UK have been published elsewhere.^18^^,^^19^ This analysis is based on monthly follow-up data to Feb 21, 2022.

For the post-primary analysis, we included all participants who had received a two-dose or three-dose primary vaccination course of either the ChAdOx1 nCoV-19 (Oxford–AstraZeneca; hereafter ChAdOx1) or BNT162b2 mRNA (Pfizer–BioNTech) vaccines. For the booster analysis, we considered all participants who had additionally received a booster dose of BNT162b2 or mRNA-1273 (Moderna). Participants were considered fully vaccinated 14 days after their second vaccine dose, if receiving a two-dose primary course, or 14 days after their third dose, if receiving a three-dose primary course (offered to immunosuppressed people in the UK^20^). Participants were considered boosted 14 days after their third vaccine dose, if receiving a two-dose primary course, or 14 days after their fourth vaccine dose, if receiving a three-dose primary course.

COVIDENCE UK is registered with ClinicalTrials.gov, NCT04330599, and was approved by Leicester South Research Ethics Committee (ref 20/EM/0117). All participants provided informed consent to participate.

### Outcomes

The primary outcome was incident SARS-CoV-2 after primary or booster vaccinations, defined as a self-reported positive result on a lateral flow or RT-PCR test for SARS-CoV-2. Time of breakthrough infection was defined as the date the test was taken.

### Independent variables

82 potential determinants of breakthrough infection were chosen a priori for inclusion in our models, covering vaccination type and timing; previous SARS-CoV-2 infection; sociodemographic, occupational, and lifestyle factors; longstanding medical conditions and prescribed medication use; Bacille Calmette Guérin vaccine status; and nutritional factors. All included factors have previously been found to be independently associated with risk of COVID-19 or pre-vaccination or post-vaccination SARS-CoV-2 antibody response.^18^^,^^19^^,^^21^ Previous infection was defined as reporting a positive result on a lateral flow or RT-PCR test for SARS-CoV-2 before start of the post-vaccination follow-up period.

When considering time-varying factors that could plausibly affect the immune response to vaccination (ie, exercise, sleep, alcohol use, smoking or vaping, anxiety, general health, use of nutritional supplements, and season of vaccination), we focused on the first vaccine dose for the post-primary analysis and on the booster dose for the post-booster analysis. We used the values from the last available monthly questionnaire before the vaccination date of interest (date of first vaccine dose, when considering post-primary outcomes, and date of booster dose, when considering post-booster outcomes). For time-varying covariates taken from monthly questionnaires that could affect SARS-CoV-2 exposure, we included all post-vaccination values observed over the follow-up period.

For post-primary outcomes, the inter-dose interval between vaccinations was calculated as the time between the first and second doses received, regardless of whether the participant was receiving a two-dose or three-dose regimen. For the post-booster analysis, the inter-dose interval was calculated as the time between the last primary course vaccination received and the booster vaccination.

To produce participant-level covariates for each class of medications investigated, questionnaire responses were mapped to drug classes listed in the British National Formulary or the DrugBank and Electronic Medicines Compendium databases if not explicitly listed in the British National Formulary, as previously described.^18^ Participants were assigned Index of Multiple Deprivation (IMD) 2019 scores, or equivalent scores for devolved administrations, according to their postcode.

### Statistical analysis

We carried out two separate analyses: a post-primary analysis and a post-booster analysis. Participants were included in the post-primary analysis when fully vaccinated, and were censored either at time of breakthrough infection, 13 days after their booster dose, or at the end of follow-up, whichever came first. Participants were included in the post-booster analysis 14 days after their booster dose, and were censored either at time of breakthrough infection or end of follow-up, whichever came first. There was no minimum follow-up time. Participants who experienced a breakthrough infection in the post-primary analysis were classified as having had a previous SARS-CoV-2 infection upon entry into the post-booster analysis.

We used Cox proportional hazards models to estimate the hazard ratios (HRs) for potential determinants of breakthrough infection after vaccination. We first estimated HRs in minimally adjusted models (adjusted for age and sex), and included all factors independently associated with breakthrough infection at the 10% significance level in fully adjusted models. We additionally adjusted for frequency of testing (ie, whether participants reported a test in every questionnaire included in the analysis) and local weekly SARS-CoV-2 incidence, using incidence rates reported for each lower-tier local authority^7^ and assigning them to participants according to their residential postcode. For participants without postcode data, we assigned them the weekly incidence of their country of residence, if available, or of the UK as a whole. We replaced missing values of time-varying covariates with mean values calculated for each participant over their follow-up period; if unavailable, we used the last value observed before vaccination.

We carried out two sensitivity analyses. First, we censored participants in the post-primary analysis at the date of their booster dose, rather than 14 days after, to exclude immediate effects of the booster dose. Second, we restricted our post-primary analysis to the period before Omicron dominance and our post-booster analysis to the period after Omicron dominance; the cutoff point for the two periods was set as Dec 15, 2021, which we estimated to be the date of Omicron dominance in the UK.^22^ We additionally adjusted for time since booster dose for participants included in the post-booster post-Omicron analysis, as some had received their booster dose before Dec 15, 2021. As an exploratory analysis, we additionally considered interactions between both age and weekly SARS-CoV-2 incidence and the other significant predictors in the models, retaining key interactions using backwards selection and comparing models using the likelihood ratio test.

We used orthogonal polynomial contrasts to test for linear trends in ordinal variables included in fully adjusted models. Correlation matrices were examined to check for collinearity between variables. The proportional hazards assumption for each model was tested using Schoenfeld residuals and visual assessment of log–log plots of survival.

Analyses were done using Stata (version 17.0).

### Role of the funding source

The study funders had no role in the study design, data analysis, data interpretation, or writing of the report.

## Results

**Figure 1:**
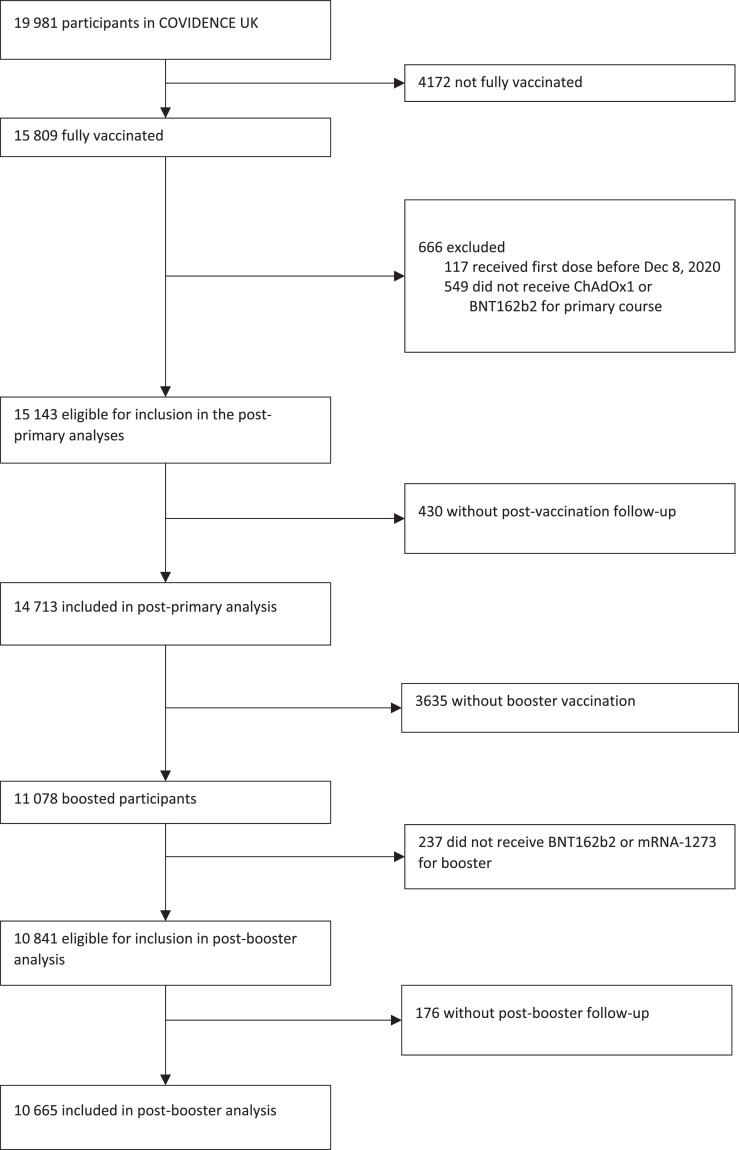
Study flow diagram. ChAdOx1 = ChAdOx1 nCoV-19.

14 713 participants were included in the post-primary analysis and 10 665 were included in the post-booster analysis (Figure 1), with a median follow-up of 203 days (IQR 195–216) in the post-primary cohort and 85 days (66–103) in the post-booster cohort (Table 1). The cohort characteristics were largely similar, with boosted participants being slightly older (Table 1). The earliest entry into the post-primary cohort was Jan 12, 2021, and the earliest entry into the post-booster cohort was Sept 5, 2021.

**Table 1:** Participant characteristics.

	Post-primary cohort (*n* = 14 713)	Post-booster cohort (*n* = 10 665)
Sociodemographic and lifestyle factors		
Age, years	62·9 (54·3–69·5)	64·3 (56·8–70·1)
<30	324 (2·2%)	124 (1·2%)
30 to <40	693 (4·7%)	328 (3·1%)
40 to <50	1464 (10·0%)	823 (7·7%)
50 to <60	3469 (23·6%)	2376 (22·3%)
60 to <70	5342 (36·3%)	4311 (40·4%)
≥70	3421 (23·3%)	2703 (25·3%)
Sex		
Female	10 430 (70·9%)	7511 (70·4%)
Male	4283 (29·1%)	3154 (29·6%)
Ethnicity*		
White	14 068 (95·6%)	10 290 (96·5%)
Mixed, multiple, or other ethnic groups	355 (2·4%)	224 (2·1%)
South Asian	212 (1·4%)	114 (1·1%)
Black, African, Caribbean, or Black British	77 (0·5%)	36 (0·3%)
Country of residence*		
England	13021 (88·8%)	9515 (89·3%)
Northern Ireland	253 (1·7%)	167 (1·6%)
Scotland	850 (5·8%)	616 (5·8%)
Wales	535 (3·6%)	363 (3·4%)
Housing*		
Owns own home	9527 (64·8%)	7535 (70·7%)
Mortgage	3418 (23·2%)	2157 (20·2%)
Privately renting	866 (5·9%)	489 (4·6%)
Renting from council	446 (3·0%)	253 (2·4%)
Other	452 (3·1%)	229 (2·1%)
Number of people per bedroom*		
<1	10 089 (69·0%)	7724 (72·9%)
1 to <2	4252 (29·1%)	2719 (25·7%)
≥2	272 (1·9%)	152 (1·4%)
Quartiles of IMD rank*		
Q4 (least deprived)	3942 (26·9%)	3000 (28·2%)
Q3	3789 (25·9%)	2838 (26·6%)
Q2	3635 (24·8%)	2577 (24·2%)
Q1 (most deprived)	3282 (22·4%)	2238 (21·0%)
Frontline worker*		
No	11 974 (81·5%)	8875 (83·3%)
Non-health or care	1464 (10·0%)	924 (8·7%)
Health or care	1261 (8·6%)	857 (8·0%)
Highest educational level attained*		
Primary or secondary	1562 (10·6%)	1090 (10·2%)
Higher or further (A levels)	2123 (14·4%)	1541 (14·5%)
College or university	6535 (44·5%)	4767 (44·7%)
Post-graduate	4477 (30·5%)	3256 (30·6%)
Smoking status		
Never-smoker	8310 (56·5%)	6111 (57·3%)
Ex-smoker	5708 (38·8%)	4128 (38·7%)
Current smoker	695 (4·7%)	426 (4·0%)
Vaping status*		
Never-vaper	13 823 (94·2%)	10 131 (95·2%)
Ex-vaper	435 (3·0%)	265 (2·5%)
Current vaper	414 (2·8%)	242 (2·3%)
Alcohol consumption, units per week		
0	4143 (28·2%)	2833 (26·6%)
1–7	5168 (35·1%)	3768 (35·3%)
8–14	2902 (19·7%)	2192 (20·6%)
≥15	2500 (17·0%)	1872 (17·6%)
Vaccination and breakthrough infections		
Primary vaccination course		
ChAdOx1	8969 (61·0%)	6537 (61·3%)
BNT162b2	5744 (39·0%)	4128 (38·7%)
Three-dose regimen	71 (0·4%)	31 (0·3%)
Inter-dose interval (first to second dose), weeks		
<6	482 (3·3%)	345 (3·2%)
6–10	5398 (36·7%)	3774 (35·4%)
>10	8833 (60·0%)	6546 (61·4%)
Booster vaccine combination		
ChAdOx1 primary plus BNT162b2 booster	..	5288 (48·8%)
ChAdOx1 primary plus mRNA-1273 booster	..	1364 (12·6%)
BNT162b2 primary plus BNT162b2 booster	..	1825 (35·3%)
BNT162b2 primary plus mRNA-1273 booster	..	371 (3·4%)
Inter-dose interval (primary to booster), weeks		
<20	..	79 (0·7%)
20–30	..	9706 (91·0%)
>30	..	880 (8·3%)
Days of follow-up	203 (195–216)	85 (66–103)
Breakthrough infection	1051 (7·1%)	1009 (9·5%)
Hospitalised	10 (1·0%)	2 (0·2%)
Days to breakthrough infection	160 (115–196)	68 (48–89)
Medical conditions		
BMI, kg/m²*		
<25	7112 (48·4%)	5229 (49·1%)
25 to <30	4747 (32·3%)	3469 (32·6%)
≥30	2830 (19·3%)	1952 (18·3%)
Arterial disease	810 (5·5%)	614 (5·8%)
Asthma	2464 (16·7%)	1726 (16·2%)
Atopy	3765 (25·6%)	2795 (26·2%)
Autoimmune disease	1361 (9·3%)	962 (9·0%)
Cancer		
Past (cured or in remission)	1334 (9·1%)	1044 (9·8%)
Present (active treatment)	134 (0·9%)	102 (1·0%)
COPD	328 (2·2%)	234 (2·2%)
Diabetes type		
Pre-diabetes	480 (3·3%)	356 (3·3%)
Diabetes	749 (5·1%)	545 (5·1%)
Heart disease	599 (4·1%)	457 (4·3%)
Hypertension	3359 (22·8%)	2560 (24·0%)
Immunodeficiency	95 (0·6%)	67 (0·6%)
Kidney disease	313 (2·1%)	248 (2·3%)
Major neurological conditions	420 (2·9%)	313 (2·9%)

Data are n (%), n/N (%), or median (IQR). BMI = body-mass index. ChAdOx1 = ChAdOx1 nCoV-19. COPD = chronic obstructive pulmonary disease. IMD = Index of Multiple Deprivation.

⁎ Missing data for ethnicity (post-primary: *n* = 1; post-booster: *n* = 1), country (*n* = 54; *n* = 4), housing (*n* = 4; *n* = 2), number of people per bedroom (*n* = 100; *n* =  70), IMD (*n* = 65; *n* = 12), frontline worker status (*n* = 14; *n* = 9), education (*n* = 16; *n* = 11), vaping (*n* = 41; *n* = 27), and BMI (*n* = 24; *n* = 15).

**Figure 2:**
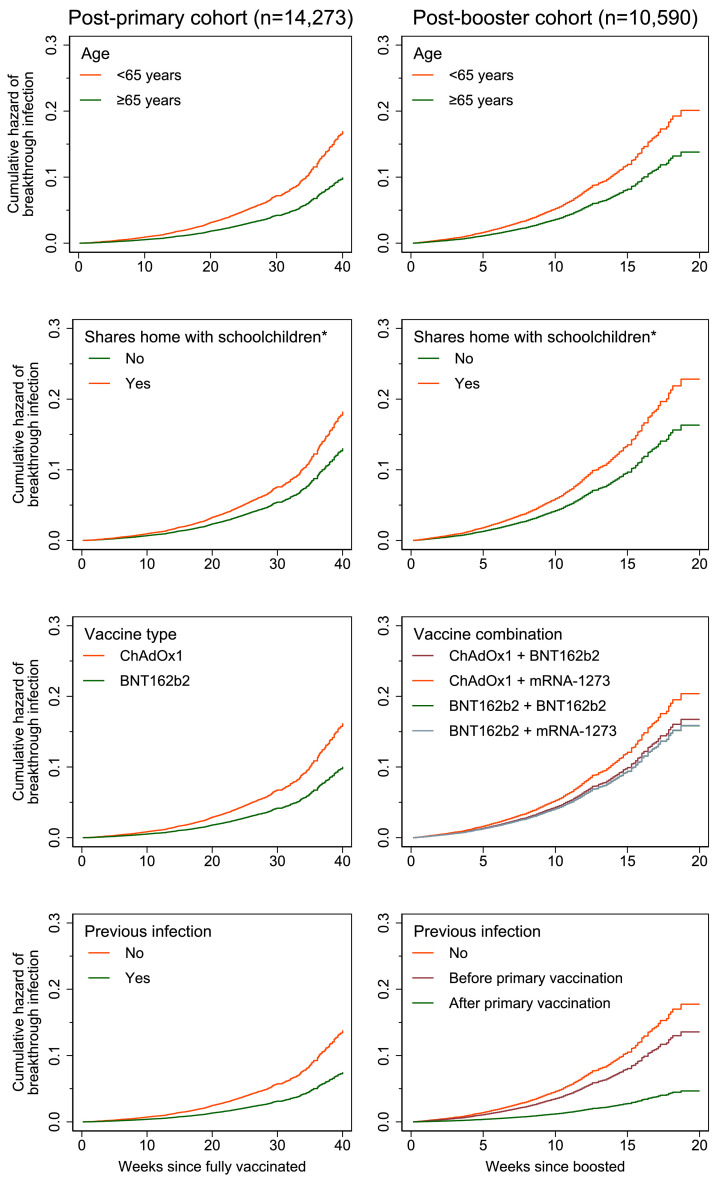
Cumulative hazard of breakthrough infection according to age, sharing a home with schoolchildren, vaccine type, and previous infection for the post-primary and post-booster cohorts. Cumulative hazards shown are from the fully adjusted models. ChAdOx1 = ChAdOx1 nCoV-19. *Aged 5–15 years.

Between Jan 12, 2021, and Feb 21, 2022, 1051 (7·1%) breakthrough infections were reported among fully vaccinated participants. After adjustment for age and sex, 24 factors were associated with risk of post-primary breakthrough infection (Table 2; see appendix Table S3 for factors for which no association was found). When included together in a fully adjusted model, we observed that primary or secondary education (*vs* postgraduate), higher number of people per bedroom (*vs* <1 person per bedroom), sharing a home with schoolchildren, pre-vaccination vaping, any visits to or from other households, frequent visits to indoor public places other than shops (*vs* no visits), greater interval between vaccine doses, a primary course of ChAdOx1 (*vs* BNT162b2), and receiving the first vaccine dose between mid-April and mid-October, 2021, were independently associated with increased risk of breakthrough infection (Table 2; Figure 2). Older age, being a frontline worker within health or social care (*vs* no frontline occupation), and having previously tested positive for SARS-CoV-2 were independently associated with reduced risk of breakthrough infection (Table 2; Figure 2).

**Table 2:** Risk factors for breakthrough SARS-CoV-2 infection in the post-primary cohort.

	Minimally adjusted model		Fully adjusted model	
	HR (95% CI)	*p* value	HR (95% CI)	*p* value
Age, years	0·95 (0·95–0·96)	<0·0001	0·97 (0·96–0·97)	<0·0001
Sex				
Female	1·00		1·00	
Male	1·10 (0·96–1·27)	0·1616	1·05 (0·90–1·21)	0·5437
Highest educational level attained				
Post-graduate	1·00		1·00	
College or university	1·06 (0·92–1·22)	0·4387	1·13 (0·98–1·32)	0·0937
Higher or further (A levels)	1·18 (0·97–1·43)	0·0922	1·23 (1·00–1·50)	0·0473
Primary or secondary	1·68 (1·37–2·06)	<0·0001	1·78 (1·44–2·20)	<0·0001
*p for linear trend*	..	..	..	<0·0001
Frontline worker				
No	1·00		1·00	
Non-health	1·32 (1·11–1·56)	0·0013	1·13 (0·95–1·35)	0·1626
Health or care	0·49 (0·39–0·61)	<0·0001	0·65 (0·51–0·84)	0·0008
Housing				
Owns own home	1·00		1·00	
Mortgage	1·23 (1·05–1·43)	0·0118	1·09 (0·92–1·29)	0·3035
Privately renting	0·87 (0·66–1·13)	0·2820	0·83 (0·63–1·09)	0·1828
Renting from council	1·46 (1·07–1·98)	0·0160	1·34 (0·95–1·89)	0·0909
Other	0·84 (0·60–1·17)	0·3077	0·87 (0·62–1·23)	0·4345
Number of people per bedroom				
<1	1·00		1·00	
1 to <2	1·32 (1·15–1·51)	<0·0001	1·15 (0·99–1·34)	0·0651
≥2	1·82 (1·33–2·48)	0·0002	1·71 (1·23–2·38)	0·0015
*p for linear trend*	..	..	..	0·0122
Multigenerational households				
Living alone	0·84 (0·70–1·01)	0·0655	0·88 (0·70–1·11)	0·2930
Single generation	1·00		1·00	
Two or more generations	1·29 (1·13–1·48)	0·0001	1·04 (0·86–1·25)	0·6808
*p for trend*	..	..	..	0·2420
Shares home with schoolchildren (5–15 years)				
No	1·00		1·00	
Yes	1·66 (1·42–1·94)	<0·0001	1·37 (1·09–1·72)	0·0061
Shares home with working-age adults (16–64 years)				
No	1·00		1·00	
Yes	1·23 (1·06–1·41)	0·0050	1·00 (0·83–1·20)	0·9893
Alcohol consumption, units per week				
0	1·00		1·00	
1–7	1·07 (0·92–1·25)	0·3489	1·05 (0·90–1·23)	0·5328
8–14	1·18 (0·99–1·40)	0·0671	1·15 (0·96–1·38)	0·1380
≥15	1·22 (1·01–1·47)	0·0441	1·17 (0·96–1·43)	0·1163
*p for linear trend*	..	..	..	0·0667
Occasional or daily vaping				
No	1·00		1·00	
Yes	1·50 (1·14–1·97)	0·0035	1·61 (1·22–2·12)	0·0008
Any visits to or from other households in past week				
No	1·00		1·00	
Yes	1·23 (1·05–1·44)	0·0103	1·19 (1·02–1·40)	0·0318
Weekly visits to shops				
0	1·00		1·00	
1	1·11 (0·80–1·54)	0·5295	1·02 (0·73–1·42)	0·9302
2–3	1·27 (0·95–1·69)	0·1130	1·15 (0·85–1·55)	0·3786
≥4	1·33 (0·99–1·77)	0·0546	1·10 (0·81–1·50)	0·5349
*p for linear trend*	..	..	..	0·4450
Weekly visits to other indoor public places (not shops)				
0	1·00		1·00	
1–2	1·19 (1·00–1·40)	0·0450	1·17 (0·99–1·40)	0·0731
≥3	1·40 (1·18–1·66)	<0·0001	1·36 (1·13–1·63)	0·0012
*p for linear trend*	..	..	..	0·0011
Weekly SARS-CoV-2 incidence (per 1000 people)	1·07 (1·06–1·08)	<0·0001	1·05 (1·03–1·06)	<0·0001
Inter-vaccine interval, weeks	1·07 (1·06–1·09)	<0·0001	1·08 (1·06–1·10)	<0·0001
Primary vaccination course				
ChAdOx1	1·78 (1·56–2·03)	<0·0001	1·63 (1·41–1·88)	<0·0001
BNT162b2	1·00		1·00	
Season of first vaccination				
Mid-October to mid-April (Winter)	1·00		1·00	
Mid-April to mid-October (Summer)	2·09 (1·70–2·57)	<0·0001	1·99 (1·59–2·49)	<0·0001
Previous infection				
No	1·00		1·00	
Yes	0·59 (0·44–0·81)	0·0009	0·54 (0·39–0·74)	0·0002
Daily portions of fruit, vegetables, and salad				
0–2	1·00		1·00	
3–4	1·16 (0·95–1·40)	0·1450	1·15 (0·94–1·40)	0·1788
5	1·12 (0·90–1·39)	0·2978	1·07 (0·86–1·34)	0·5507
≥6	1·21 (0·99–1·47)	0·0583	1·20 (0·98–1·47)	0·0766
*p for linear trend*	..	..	..	0·1598
General health				
Excellent	1·00		1·00	
Very good	0·85 (0·72–1·00)	0·0501	0·86 (0·73–1·02)	0·0928
Good	0·89 (0·74–1·07)	0·2047	0·95 (0·79–1·15)	0·6124
Fair	0·99 (0·79–1·23)	0·9330	1·11 (0·88–1·41)	0·3609
Poor	0·73 (0·51–1·03)	0·0757	0·94 (0·64–1·37)	0·7402
*p for trend*	..	..	..	0·3232
Hypertension				
No	1·00		1·00	
Yes	0·86 (0·72–1·02)	0·0887	0·98 (0·80–1·19)	0·8077
Immunodeficiency				
No	1·00		1·00	
Yes	0·14 (0·02–0·99)	0·0489	0·15 (0·02–1·10)	0·0625
Calcium channel blockers				
No	1·00		1·00	
Yes	0·80 (0·62–1·03)	0·0799	0·85 (0·63–1·14)	0·2852
Anticholinergics				
No	1·00		1·00	
Yes	0·69 (0·50–0·94)	0·0186	0·73 (0·52–1·01)	0·0602
Reported COVID-19 test in every questionnaire				
No	1·00		1·00	
Yes	1·98 (1·72–2·27)	<0·0001	2·04 (1·77–2·35)	<0·0001

Risk factors associated with breakthrough infection at the 10% significance level in minimally adjusted models (ie, adjusted for age and sex) are shown. The fully adjusted analysis included all shown risk factors and was done in 14 273 participants, with 1022 breakthrough infections. *p* values for linear trends are shown; values for quadratic, cubic, and quartic trends (where applicable) are shown in the appendix (Table S5). ChAdOx1 = ChAdOx1 nCoV-19. HR = hazard ratio.

Between Sept 5, 2021, and Feb 21, 2022, 1009 (9·5%) breakthrough infections were reported among boosted participants, the majority (948 [94·0%]) of which occurred after the Omicron variant became dominant in the UK. After adjustment for age and sex, 31 factors were associated with risk of post-booster breakthrough infection (Table 3; see appendix Table S4 for factors for which no association was found). When included together in a fully adjusted model, we observed that lower levels of education (*vs* postgraduate), having a mortgage or privately renting (*vs* owning own home), sharing a home with schoolchildren or working-age adults, high levels of pre-vaccination alcohol consumption (*vs* none), frequent visits to indoor public places other than shops (*vs* none), and a primary course of ChAdOx1 followed by an mRNA-1273 booster (*vs* BNT162b2 for both primary course and booster) were associated with increased risk of breakthrough infection, whereas older age; mixed or South Asian ethnicity (*vs* White); an average of no more than 5 hours of sleep pre-vaccination (*vs* 7 hours); receiving the booster vaccination between mid-April and mid-October; having had a previous infection after the primary course of vaccination; and taking fish oil, krill oil, or other omega-3 supplements were associated with reduced risk (Table 3; Figure 2). Increased local weekly SARS-CoV-2 incidence and high frequency of testing was associated with increased risk in all models (Tables 2, 3).

**Table 3:** Risk factors for breakthrough SARS-CoV-2 infection in the post-booster cohort.

	Minimally adjusted		Fully adjusted	
	HR (95% CI)	*p* value	HR (95% CI)	*p* value
Age, years	0·96 (0·96–0·97)	<0·0001	0·97 (0·97–0·98)	<0·0001
Sex				
Female	1·00		1·00	
Male	0·93 (0·80–1·07)	0·3218	0·86 (0·74–1·00)	0·0517
Ethnicity				
White	1·00		1·00	
Mixed, multiple, or other ethnic groups	0·27 (0·13–0·54)	0·0002	0·28 (0·14–0·57)	0·0004
South Asian	0·40 (0·19–0·85)	0·0174	0·46 (0·22–0·97)	0·0427
Black, African, Caribbean, or Black British	0·98 (0·37–2·62)	0·9699	0·95 (0·35–2·56)	0·9219
Highest educational level attained				
Post-graduate	1·00		1·00	
College or university	1·06 (0·92–1·23)	0·4096	1·09 (0·94–1·27)	0·2493
Higher or further (A levels)	1·19 (0·98–1·45)	0·0805	1·30 (1·06–1·59)	0·0101
Primary or secondary	1·30 (1·04–1·63)	0·0210	1·46 (1·16–1·83)	0·0014
*p for trend*	..	..	..	0·0003
Frontline worker				
No	1·00		1·00	
Non-health	1·20 (0·98–1·47)	0·0823	1·05 (0·86–1·30)	0·6193
Health or care	0·74 (0·60–0·91)	0·0043	0·90 (0·71–1·15)	0·4189
Housing				
Owns own home	1·00		1·00	
Mortgage	1·23 (1·04–1·45)	0·0137	1·21 (1·03–1·44)	0·0239
Privately renting	1·27 (0·97–1·66)	0·0864	1·47 (1·11–1·95)	0·0071
Renting from council	0·93 (0·60–1·44)	0·7454	1·13 (0·72–1·78)	0·5820
Other	0·92 (0·60–1·39)	0·6819	1·14 (0·75–1·75)	0·5373
Multigenerational households				
Living alone	0·78 (0·65–0·94)	0·0080	0·90 (0·73–1·12)	0·3465
Single generation	1·00		1·00	
Two or more generations	1·15 (0·99–1·32)	0·0615	0·98 (0·82–1·17)	0·7852
*p for trend*	..	..	..	0·5742
Shares home with schoolchildren (5–15 years)				
No	1·00		1·00	
Yes	1·45 (1·19–1·76)	0·0002	1·35 (1·06–1·74)	0·0168
Shares home with working-age adults (16–64 years)				
No	1·00		1·00	
Yes	1·38 (1·19–1·59)	<0·0001	1·27 (1·06–1·50)	0·0078
Alcohol consumption, units per week				
0	1·00		1·00	
1–7	1·19 (1·01–1·40)	0·0391	1·04 (0·88–1·23)	0·6465
8–14	1·21 (1·00–1·45)	0·0502	0·98 (0·81–1·19)	0·8457
≥15	1·53 (1·27–1·83)	<0·0001	1·26 (1·04–1·53)	0·0173
*p for trend*	..	..	..	0·0584
Travelled outside of the UK since last questionnaire				
No	1·00		1·00	
Yes	1·24 (1·06–1·44)	0·0073	1·07 (0·91–1·26)	0·3848
Weekly journeys on public transport				
0	1·00		1·00	
1–5	1·09 (0·94–1·28)	0·2516	1·00 (0·85–1·17)	0·9628
≥6	1·27 (1·02–1·58)	0·0346	1·16 (0·91–1·46)	0·2264
*p for trend*	..	..	..	0·4105
Any visits to or from other households in past week				
No	1·00		1·00	
Yes	1·16 (0·98–1·36)	0·0837	1·07 (0·91–1·26)	0·4252
Weekly visits to shops				
0	1·00		1·00	
1	0·94 (0·68–1·31)	0·7216	0·91 (0·65–1·27)	0·5782
2–3	1·12 (0·84–1·49)	0·4335	1·04 (0·78–1·40)	0·7740
≥4	1·40 (1·06–1·85)	0·0196	1·18 (0·88–1·59)	0·2779
*p for trend*	..	..	..	0·0258
Weekly visits to other indoor public places (not shops)				
0	1·00		1·00	
1–2	1·18 (0·99–1·40)	0·0663	1·08 (0·91–1·30)	0·3732
≥3	1·59 (1·33–1·89)	<0·0001	1·29 (1·07–1·56)	0·0076
*p for trend*	..	..	..	0·0049
Vigorous physical exercise, h per week				
0	0·84 (0·71–0·99)	0·0373	0·89 (0·75–1·06)	0·1918
1	0·92 (0·78–1·07)	0·2671	0·89 (0·76–1·05)	0·1633
2	1·00		1·00	
*p for trend*	..	..	..	0·2250
Actual sleep, h per night				
≤5	0·71 (0·56–0·90)	0·0050	0·75 (0·59–0·96)	0·0198
6	1·01 (0·87–1·17)	0·9218	1·01 (0·87–1·17)	0·8863
7	1·00		1·00	
≥8	0·98 (0·83–1·16)	0·7998	1·03 (0·87–1·22)	0·7260
*p for trend*	..	..	..	0·0555
Weekly SARS-CoV-2 incidence	1·09 (1·08–1·10)	<0·0001	1·09 (1·08–1·10)	<0·0001
Inter-vaccine interval (primary to booster), weeks	0·97 (0·95–0·99)	0·0077	0·98 (0·96–1·01)	0·2029
Combination of primary and booster vaccinations				
ChAdOx1 plus BNT162b2 booster	1·16 (1·01–1·33)	0·0341	1·06 (0·91–1·23)	0·4558
ChAdOx1 plus mRNA-1273 booster	1·41 (1·14–1·74)	0·0014	1·26 (1·00–1·57)	0·0459
BNT162b2 plus BNT162b2 booster	1·00		1·00	
BNT162b2 plus mRNA-1273 booster	1·07 (0·73–1·56)	0·7268	0·99 (0·67–1·46)	0·9663
Season of booster vaccination				
Mid-October to mid-April (Winter)	1·00		1·00	
Mid-April to mid-October (Summer)	0·63 (0·53–0·74)	<0·0001	0·56 (0·46–0·68)	<0·0001
Previous infection				
No evidence of previous infection	1·00		1·00	
Previous infection before primary course of vaccination	0·87 (0·64–1·17)	0·3603	0·75 (0·55–1·01)	0·0594
Previous infection after primary course of vaccination	0·30 (0·18–0·52)	<0·0001	0·28 (0·16–0·47)	<0·0001
Probiotics				
No	1·00		1·00	
Yes	0·77 (0·56–1·05)	0·0948	0·79 (0·57–1·09)	0·1524
Fish oil, krill oil, or other omega-3 supplements				
No	1·00		1·00	
Yes	0·79 (0·64–0·99)	0·0409	0·80 (0·63–1·00)	0·0481
Cod liver oil supplements				
No	1·00		1·00	
Yes	0·78 (0·60–1·02)	0·0744	0·80 (0·61–1·04)	0·0966
Atopy				
No	1·00		1·00	
Yes	0·85 (0·74–0·98)	0·0258	0·89 (0·77–1·03)	0·1154
Diabetes				
No diabetes	1·00		1·00	
Pre-diabetes	0·74 (0·48–1·12)	0·1563	0·84 (0·55–1·29)	0·4283
Type 1 diabetes	0·96 (0·48–1·93)	0·9085	0·88 (0·44–1·78)	0·7239
Type 2 diabetes	0·68 (0·47–0·98)	0·0390	0·89 (0·51–1·53)	0·6641
Beta blockers				
No	1·00		1·00	
Yes	0·74 (0·56–0·98)	0·0342	0·86 (0·65–1·15)	0·3164
ACE inhibitors				
No	1·00		1·00	
Yes	0·82 (0·65–1·03)	0·0915	0·98 (0·77–1·25)	0·8608
Angiotensin receptor blockers				
No	1·00		1·00	
Yes	0·76 (0·57–1·03)	0·0745	0·93 (0·69–1·27)	0·6627
Thiazides				
No	1·00		1·00	
Yes	0·51 (0·32–0·83)	0·0067	0·62 (0·38–1·03)	0·0654
Metformin				
No	1·00		1·00	
Yes	0·62 (0·39–0·99)	0·0454	0·87 (0·44–1·72)	0·6844
Reported COVID-19 test in every questionnaire				
No	1·00		1·00	
Yes	2·11 (1·86–2·39)	<0·0001	1·97 (1·74–2·24)	<0·0001

Risk factors associated with breakthrough infection at the 10% significance level in minimally adjusted models (ie, adjusted for age and sex) are shown. The fully adjusted analysis included all shown risk factors and was done in 10 590 participants, with 997 breakthrough infections. *p* values for linear trend are shown; values for quadratic, cubic, and quartic trends (where applicable) are shown in the appendix (Table S5). ACE = angiotensin-converting enzyme. ChAdOx1 = ChAdOx1 nCoV-19. HR = hazard ratio.

In the fully adjusted models, there was evidence of violation of the proportional hazards assumption for primary vaccination course (*p* = 0·047) and COVID-19 testing frequency (*p* = 0·003) in the post-primary model and for probiotics (*p* = 0·046) and angiotensin receptor blockers (*p* = 0·023) in the post-booster model. Stratifying by these variables did not substantially affect our estimates (appendix Tables S6, S7). Examination of correlation matrices of the fitted models showed little evidence of collinearity, with the strongest correlations between presence of schoolchildren at home and multigenerational households, and between metformin use and diabetes (appendix Figures S4, S5). Exclusion of the schoolchildren variable in the post-primary analysis led to households of at least two generations being at significantly increased risk of breakthrough infection (appendix Table S8); however, estimates for other variables were not affected and inclusion of the schoolchildren variable was supported by the likelihood ratio test (appendix Table S8). Exclusion of the schoolchildren and metformin variables in the post-booster analysis had no effect on our estimates (Table S9).

Censoring participants in the post-primary analysis at their booster date led to the inclusion of IMD rank in the fully adjusted model, along with small changes in the estimates for educational attainment, housing, and visits to or from other households (appendix Table S10). Restricting the post-primary analysis to the pre-Omicron period led to the inclusion of IMD rank, hours of sleep, ACE inhibitors, and sodium-glucose co-transporter-2 (SGLT2) inhibitors in the fully adjusted model, along with the exclusion of number of visits to the shops and immunodeficiency (appendix Table S11). Of the newly included variables, only SGLT2 inhibitors were associated with increased risk of breakthrough infection in the fully adjusted model. The strength of association increased for schoolchildren and number of people per bedroom, and was inverted for weekly SARS-CoV-2 incidence (appendix Table S11), likely reflecting changes in behaviour in the face of the approaching Omicron wave. Restricting the post-booster analysis to the post-Omicron period led to the inclusion of general health in the fully adjusted model, which was not found to be associated with risk of breakthrough infection, and the exclusion of educational attainment, frontline worker status, inter-vaccine interval, diabetes, and use of beta blockers, ACE inhibitors, and angiotensin receptor blockers (appendix Table S12). The associations with risk of breakthrough infection observed in the main model for sleep; combination of primary and booster vaccines; fish oil, krill oil, or other omega-3 supplements; and season of vaccination were not present in the restricted model, whereas use of thiazides was found to be protective (appendix Table S12).

Inclusion of interactions of significant predictors with age and weekly SARS-CoV-2 incidence led to improved model fit for both the post-primary and post-booster models (appendix Tables S13, S14). In the post-primary analysis, significant interactions were observed between age and number of people per bedroom (increasing risk with increasing age), weekly SARS-CoV-2 incidence and a ChAdOx1 primary vaccination course (decreasing risk with increasing incidence), and weekly SARS-CoV-2 incidence and sharing a home with schoolchildren (decreasing risk with increasing incidence; appendix Table S13). The main effects for these factors remained significant, and estimated effects for other factors were unaffected. In the post-booster analysis, significant interactions were observed between age and weekly visits to indoor public places other than shops (increasing risk with increasing age) and between age and a ChAdOx1 primary course plus mRNA-1273 booster (decreasing risk with increasing age; appendix Table S14). The main effect for a ChAdOx1 primary course plus mRNA-1273 booster was no longer significant, and estimated effects for other factors were unaffected.

## Discussion

In this large, prospective, national observational study, we found that lower levels of education, sharing a home with schoolchildren, and frequent visits to indoor public places other than shops were associated with increased risk of breakthrough SARS-CoV-2 infection, both after the primary course of vaccination and after receiving a booster dose. Older age and a previous SARS-CoV-2 infection were consistently associated with lower risk of breakthrough infection. We found higher risk of breakthrough infection after a ChAdOx1 primary course compared with a BNT162b2 primary course; however, this increased risk did not remain among ChAdOx1 recipients after a booster vaccination.

Our study shows how the risk factors for SARS-CoV-2 infection after primary or booster vaccination can differ to those in unvaccinated populations. In pre-vaccination studies, high body-mass index (BMI),^23^ Asian ethnicity,^24^ and working in a healthcare setting^25^ have all been found to be risk factors for SARS-CoV-2 infection and COVID-19, including in the same COVIDENCE UK cohort studied here.^18^ By contrast, we observed no association with BMI, either after primary or booster vaccinations, and a reduced risk of breakthrough infection among healthcare workers after their primary vaccination. This finding may reflect healthcare workers being more likely to have experienced severe infections earlier in the pandemic,^26^ which has been shown to be associated with greater antibody persistence than asymptomatic or mild infection.^27^ Notably, this protective effect was no longer present in the post-booster analysis that predominantly featured infections with the Omicron variant, which has been found to evade immunity from previous infection.^28^ While underpowered to investigate ethnicity, previous studies on our cohort have consistently shown increased risk of pre-vaccination infection among South Asian participants,^18^^,^^19^ which has not remained after vaccination. Indeed, after a booster vaccination, we observed lower risk of breakthrough infection for participants of South Asian or mixed ethnicity compared with White participants after adjusting for previous infection. This supports both pre-vaccination and post-vaccination serology findings from the same cohort, showing higher antibody titres from natural infection and from vaccination in these ethnic groups than in White participants, independently of pre-vaccination serostatus^21^ and disease severity.^19^ Few studies to date have observed associations between ethnicity and breakthrough SARS-CoV-2 infection,^14^ although lack of diversity in vaccine clinical trials^29^ and missing data in some of the larger observational studies^2^^,^^9^ have hindered investigation. Large-scale studies with a more detailed breakdown of ethnicity are needed to understand the effects of booster vaccinations in ethnic subgroups.

Our findings highlight the important role that lifestyle and behaviours play in SARS-CoV-2 transmission after vaccination. Exposure to other people, both at home and in indoor public places, remained a strong predictor of breakthrough infection for fully vaccinated and boosted participants after adjusting for weekly SARS-CoV-2 incidence. In particular, sharing a home with schoolchildren—who represented a largely unvaccinated population for much of the study period—was consistently associated with a 35% increase in risk of infection; as SARS-CoV-2 incidence grew, this effect was diminished, presumably as infection rates increased in older age groups as well. Notably, frequency of public transport use and visits to shops did not predict breakthrough infection in either model, suggesting that transmission largely occurs in settings of prolonged exposure, or where people are less likely to use masks, such as restaurants or bars. While we used pre-vaccination values for behaviours such as alcohol consumption and vaping to capture potential effects on immunogenicity,^30^ these values are unlikely to have changed greatly over follow-up and so may also reflect post-vaccination SARS-CoV-2 exposures, such as sharing of e-cigarettes^31^ or social drinking. Indeed, our finding of increased risk of breakthrough infection for participants with high levels of alcohol consumption in the post-booster analysis, but not in the post-primary analysis, suggests we were capturing post-vaccination SARS-CoV-2 exposures—particularly as the post-booster analysis was restricted to colder months, when social events were more likely to be held indoors.

Previous studies have found that lower educational attainment increases risk of SARS-CoV-2 infection^25^ and severe disease^26^ before vaccination, and our study shows that this risk remains after primary and booster vaccinations. Notably, we found no correlation between educational attainment and factors affecting SARS-CoV-2 exposure, such as number of people per bedroom or frequency of visits to other indoor public places. It is therefore unclear whether this continued increased risk reflects socioeconomic effects of low educational attainment that our study was unable to capture, or limitations in health literacy, in a landscape where public health messages changed frequently.

By contrast with studies on severe COVID-19 after vaccination,^16^^,^^17^ we found no indication of increased risk of breakthrough infection among people with comorbidities or among older participants, instead observing an inverse relationship with age. This finding is supported by other UK studies^2^^,^^32^ and matches the age patterns in infection rates observed earlier in 2022 in the UK.^33^ The fact that age remained a strong predictor in our cohort despite adjusting for behaviours such as public transport journeys and visits to indoor public places—and despite our finding that older age increased the risk posed by such risk factors—highlights the extent to which older adults, who are more likely to experience severe outcomes if infected,^34^ may still be reducing their social contacts compared with their younger counterparts.^35^ Similarly, the weak association seen between immunodeficiency and reduced risk of breakthrough infection in the post-primary cohort is likely to reflect more risk-averse behaviour in this group. These results suggest that despite adjusting for behaviours, we have not been able to capture them fully, and thus unmeasured or residual confounding may be operating in our study.

In our post-primary cohort, vaccine type was a strong predictor of breakthrough infection, with the weaker performance of ChAdOx1 mirroring studies on vaccine effectiveness in the general population^32^ and on post-vaccination serology.^21^ However, this difference seems to have been eradicated in our cohort after booster vaccination with BNT162b2. This may reflect both increased immunogenicity after boosting with BNT162b2, regardless of the primary vaccination course,^36^ and reduced effectiveness of BNT162b2 against the Omicron variant,^5^ which became dominant in the UK in mid-December, 2021. This is supported by our finding that the difference between ChAdOx1 and BNT162b2 diminished with increased SARS-CoV-2 incidence, as the period of highest incidence in our dataset was due to the Omicron wave. Although our initial results suggested that an mRNA-1273 booster might be less effective among ChAdOx1 recipients than a BNT162b2 booster, this association was lost in sensitivity and exploratory analyses. Future studies with longer follow-up will be needed to understand the long-term efficacies of the different booster vaccines among ChAdOx1 and BNT162b2 recipients.

We investigated several factors known to affect immune response to vaccination, such as season of vaccination,^37^ the inter-dose vaccine interval,^38^ and behaviours around the time of vaccination.^30^ We observed strong but inconsistent results for season of vaccination, with a higher risk of breakthrough infection among participants who received their first vaccine dose in the summer months (mid-April to mid-October) but lower risk among participants who received their booster dose in the summer months. Notably, the vast majority of first vaccinations in our cohort (14 353 [94·7%]) took place in the winter months, which is likely to have affected our results. It is likely that our season variable is additionally capturing time-varying factors not covered by our incidence and behavioural variables, such as more nuanced changes in behaviour owing to periods of restrictions and public health messaging, as well as changes in transmission risk arising from the emergence of new variants. Our finding of reduced risk of post-booster infection when taking fish oil, krill oil, or other omega-3 supplements supports results from another study;^39^ however, we observed no protective effect in the post-primary analysis or in the post-booster analysis when restricted to the period of Omicron dominance. Similarly, our finding of reduced risk of breakthrough infection in participants with low sleep duration was only present in our post-booster analysis, and goes against the understanding that sleep duration correlates positively with immune function.^40^ These results should therefore be interpreted with caution, and need to be replicated in different populations before the possibility of type 1 error can be discounted.

By contrast with studies showing that a longer inter-dose interval increases immunogenicity,^21^^,^^38^^,^^41^ we found a strong association between longer interval between first and second vaccine doses and increased risk of breakthrough infection. While a correlate of protection that translates immunogenicity findings into real-world protection has yet to be found, some preliminary studies suggest a longer interval is indeed protective.^42^ It is therefore possible that our finding of increased risk associated with longer inter-dose interval is the result of unmeasured confounding.

This study has several strengths. The size of the study, its population-based nature, and our inclusion of three of the most widely used vaccines for primary and booster vaccinations together increase the generalisability of our findings. The granular nature of the data used has allowed us to investigate a wide range of risk factors for breakthrough infection, while adjusting for weekly local SARS-CoV-2 incidence and testing behaviours. Our use of monthly follow-up data means that we have been able to adjust for changing behaviours, rather than relying on baseline values that are unlikely to be representative of behaviours across different phases of the pandemic. Use of survey data has also allowed us to capture episodes of milder disease than those included in hospital-based studies, providing a clearer picture of post-vaccination transmission in the general population. Finally, by focusing on the same cohort as previous studies that have considered risk factors of infection in a largely unvaccinated population,^18^^,^^19^ this study allows a clear comparison of pre-vaccination and post-vaccination risk factors in the same population over different periods of the pandemic.

This study also has some limitations. First, we were unable to identify the strain of the infections, meaning that we could not dissect differences between the variants that were dominant at different points in our study. To mitigate this, we present sensitivity analyses that restrict the post-primary and post-booster analyses to the periods before Omicron dominance and after Omicron dominance, respectively; however, we did not have sufficient power to conduct a post-Omicron post-primary analysis or a pre-Omicron post-booster analysis, owing to the timing of the booster rollout in the UK, which was accelerated by the emergence of Omicron. Second, with very few hospitalisations, we did not have the power to assess risk factors for different levels of disease severity in vaccinated individuals. Third, we relied on self-reported test results, both for our primary outcome and to define previous infections, meaning we are likely to have missed some asymptomatic or mild infections. Additionally, self-testing behaviour will have varied both between participants and over the study period, which could affect the reliability of our results. However, we have adjusted for differences in testing behaviour by including a variable measuring whether or not a participant reported a test for every monthly observation included in the analysis. Furthermore, the UK's offering of twice-weekly free testing for the majority of our study period^43^ should have provided participants with equal opportunities to test, whether symptomatic or asymptomatic. Fourth, as a self-selected cohort, several groups—including people younger than 30 years, people of lower socioeconomic status, and non-White ethnic groups—are under-represented in COVIDENCE UK, which can limit generalisability and lead to collider bias. However, lack of representativeness is not necessarily a barrier to valid scientific inference,^44^ and by considering interactions and correlations between covariates, and conducting several sensitivity analyses, we have taken steps to test the robustness of our results. Fifth, as with any observational study, it is possible that some of the associations we report can be explained by residual or unmeasured confounding; we have attempted to minimise this by adjusting for a comprehensive range of potential risk factors for infection, made possible by the large number of breakthrough infections recorded in our study (ie, >30 events per variable in each fully adjusted model). Finally, we explored several potential associations and cannot exclude the possibility that some achieved statistical significance as a result of type 1 error; however, by including the same predictors in post-primary and post-booster analyses, and presenting p-for-trend analyses for ordinal variables, we hope to have given readers the tools for a careful interpretation of our results.

In conclusion, primary and booster vaccinations have changed the landscape of the SARS-CoV-2 pandemic, attenuating the effects of risk factors for COVID-19 in unvaccinated populations, such as Asian ethnicity and BMI. We observed a clear difference between the efficacies of ChAdOx1 and BNT162b2 in fully vaccinated individuals, but the combination of booster vaccinations and the Omicron variant appears to have levelled the playing field. Key determinants of breakthrough infection remaining both after primary and booster vaccinations are therefore behaviours and lifestyles that affect individuals' exposure to other people. As countries increasingly remove public health restrictions, we are truly entering an era of personal responsibility.

## Contributors

A.R.M. wrote the study protocol, with input from H.H., M.T., and S.O.S. H.H., M.T., G.A.D., R.A.L., C.J.G., F.K., A.S., and A.R.M. contributed to questionnaire development and design. H.H. co-ordinated and managed the study, with input from A.R.M., D.A.J., M.T., and S.O.S. H.H., A.R.M., and S.O.S. supported recruitment. G.V., F.T., M.T., H.H., and D.A.J. contributed to data management and coding medication data. G.V. and F.T. directly accessed and verified the data. Statistical analyses were done by G.V., with input from A.R.M. and F.T. G.V. wrote the first draft of the report, with input from A.R.M. All authors read and approved the final manuscript. G.V., D.A.J., H.H., F.T., M.T., S.O.S., and A.R.M. had full access to all data in the study, and all authors had final responsibility for the decision to submit for publication.

## Data sharing statement

De-identified participant data will be made available upon reasonable request to the corresponding authors.

## Declaration of interests

R.A.L. has received grants from UKRI Medical Research Council, UKRI Economic and Social Research Council, Health Data Research UK, and Health and Care Research Wales. R.A.L. is a member of the Welsh Government COVID-19 Technical Advisory group, in an unremunerated role. A.S. is a member of the Scottish Government's Standing Committee on Pandemics, the Scottish Science Advisory Council, the UK Government's New and Emerging Respiratory Virus Threats Risk Stratification Subgroup and the Department of Health and Social Care's COVID-19 Therapeutics Modelling Group. He was a member of the Scottish Government Chief Medical Officer's COVID-19 Advisory Group and AstraZeneca's Thrombotic Thrombocytopenic Taskforce. All of A.S.’ roles are unremunerated. A.R.M. declares receipt of funding to support vitamin D research from the following companies who manufacture or sell vitamin D supplements: Pharma Nord DSM Nutritional Products, Thornton & Ross, and Hyphens Pharma. A.R.M. also declares support for attending meetings from the following companies who manufacture or sell vitamin D supplements: Pharma Nord and Abiogen Pharma. A.R.M. also declares participation on the Data and Safety Monitoring Board for the Chair, DSMB, VITALITY trial (Vitamin D for Adolescents with HIV to reduce musculoskeletal morbidity and immunopathology). A.R.M. also declares unpaid work as a Programme Committee member for the Vitamin D Workshop. A.R.M. also declares receipt of vitamin D capsules for clinical trial use from Pharma Nord, Synergy Biologics, and Cytoplan. All other authors declare no competing interests.

## Appendix Supplementary materials

Image, application 1
