# Supplementary material for: Risk factors for SARS-CoV-2 infection after primary vaccination with ChAdOx1 nCoV-19 or BNT162b2 and after booster vaccination with BNT162b2 or mRNA-1273: A population-based cohort study (COVIDENCE UK)
Source: Lancet Reg Health Eur. 2022 Sep 23;22:100501. doi: 10.1016/j.lanepe.2022.100501 (PMC9499825; doi:10.1016/j.lanepe.2022.100501)
Supplement: Supplementary file 1 [file mmc1.docx]

**Supplementary material**

**Risk factors for SARS-CoV-2 infection after primary vaccination with ChAdOx1 nCoV-19 or BNT162b2 and after booster vaccination with BNT162b2 or mRNA-1273:** **a population-based cohort study (COVIDENCE UK)**

Giulia Vivaldi, David A Jolliffe, Hayley Holt, Florence Tydeman, Mohammad Talaei, Gwyneth A Davies, Ronan A Lyons, Christopher J Griffiths, Frank Kee, Aziz Sheikh, Seif O Shaheen, Adrian R Martineau

**Correspondence to: Giulia Vivaldi and Adrian R Martineau**

Blizard Institute, Barts and The London School of Medicine and Dentistry,
Queen Mary University of London, 4 Newark St, London E1 2AT, UK
Email: [g.vivaldi@qmul.ac.uk](mailto:g.vivaldi@qmul.ac.uk) or [a.martineau@qmul.ac.uk](mailto:a.martineau@qmul.ac.uk)

**Table of contents**

[***Figure S1:* Geographical distribution of participants** 3](#_Toc112158831)

[***Table S1*: Baseline questions** 4](#_Toc112158832)

[***Table S2:* Monthly follow-up questions** 12](#_Toc112158833)

[***Figure S2:* Correlation matrix of raw variables: post-primary cohort** 15](#_Toc112158834)

[***Figure S3:* Correlation matrix of raw variables: post-booster cohort** 16](#_Toc112158835)

[***Figure S4:* Correlation matrix of the estimators: assessment of multicollinearity for the post-primary analysis** 17](#_Toc112158836)

[***Figure S5:* Correlation matrix of the estimators: assessment of multicollinearity for the post-booster analysis** 18](#_Toc112158837)

[***Table S3:* Factors not associated with breakthrough SARS-CoV-2 infection in the post-primary cohort, after adjustment for age and sex** 19](#_Toc112158838)

[***Table S4:* Factors not associated with breakthrough SARS-CoV-2 infection in the post-booster cohort, after adjustment for age and sex** 21](#_Toc112158839)

[***Table S5:* Trend analysis showing tests for linear, quadratic, cubic, and quartic trends of ordinal variables** 23](#_Toc112158840)

[***Table S6:* Risk factors for breakthrough SARS-CoV-2 infection in the pre-booster cohort, stratified by primary vaccination course and COVID-19 test frequency** 24](#_Toc112158841)

[***Table S7:* Risk factors for breakthrough infection in the post-booster cohort, stratified by probiotics and angiotensin receptor blockers** 26](#_Toc112158842)

[***Table S8:* Risk factors for breakthrough SARS-CoV-2 infection in the post-primary cohort, omitting schoolchildren variable** 29](#_Toc112158843)

[***Table S9:* Risk factors for breakthrough SARS-CoV-2 infection in the post-booster cohort, omitting schoolchildren and metformin variables** 31](#_Toc112158844)

[***Table S10:* Sensitivity analysis: Risk factors for breakthrough SARS-CoV-2 infection in the post-primary cohort, censored at date of booster dose** 34](#_Toc112158845)

[***Table S11:* Sensitivity analysis: Risk factors for breakthrough SARS-CoV-2 infection in the post-primary cohort (pre-Omicron)** 36](#_Toc112158846)

[***Table S12:* Sensitivity analysis: Risk factors for breakthrough SARS-CoV-2 infection in the post-booster cohort (post-Omicron)** 38](#_Toc112158847)

[***Table S13:* Exploratory analysis: Risk factors for breakthrough SARS-CoV-2 infection in the post-primary cohort, including interactions for age and weekly SARS-CoV-2 incidence** 40](#_Toc112158848)

[***Table S14:* Exploratory analysis: Risk factors for breakthrough SARS-CoV-2 infection in the post-booster cohort, including interactions for age and weekly SARS-CoV-2 incidence** 42](#_Toc112158849)

[***Table S15:* Missing data** 45](#_Toc112158850)

[***Table S16:* Monthly response rates** 46](#_Toc112158851)

# ***Figure S1:* Geographical distribution of participants**

***
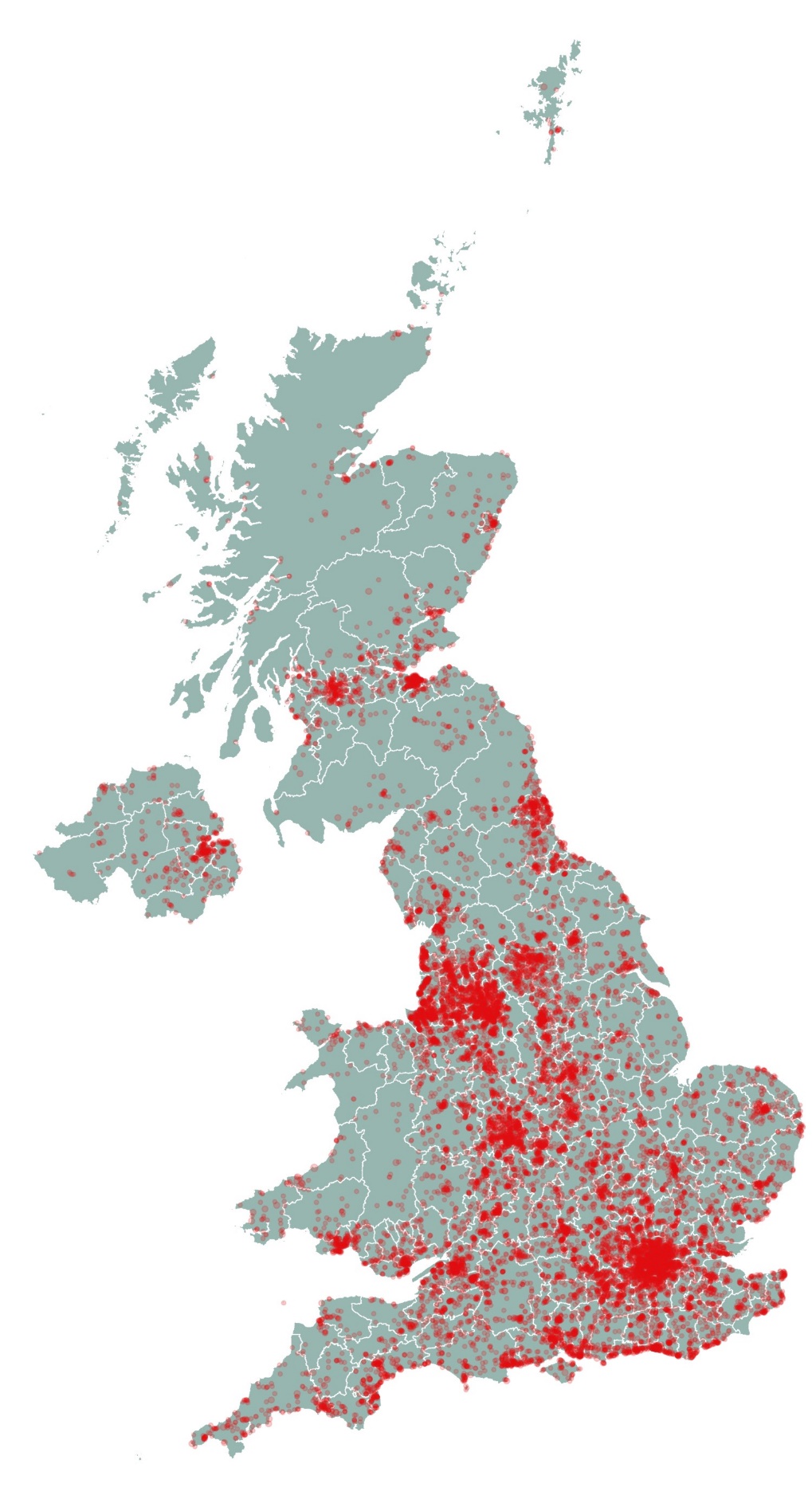
***Participants are mapped by postcode; the size of the points are relative to the number of people living at that postcode.

# ***Table S1*: Baseline questions**

| **Sociodemographic** | | | | | | | | | | | | |
| --- | --- | --- | --- | --- | --- | --- | --- | --- | --- | --- | --- | --- |
| Date of birth (DD/MM/YYYY) |  | | | | | | | | | | | |
| Post code |  | | | | | | | | | | | |
| Address |  | | | | | | | | | | | |
| Please state your **assigned sex at birth.** | • Male  • Female | | | | | | | | | | | |
| What is your ethnic origin? | • White   - English / Welsh / Scottish / Northern Irish / British - Irish - Gypsy or Irish Traveller - Any other white background   • Mixed / Multiple ethnic groups   - White and Black Caribbean - White and Black African - White and Asian - Any other Mixed / Multiple ethnic backgrounds   • Asian / Asian British   - Indian - Pakistani - Bangladeshi - Chinese - Any other Asian background   • Black / African / Caribbean / Black British   - African - Caribbean - Any other Black / African / Caribbean background   • Arab  • Other Ethnic Group | | | | | | | | | | | |
| Were you born in the UK? | • Yes  • No | | | | | | | | | | | |
| Are you a ‘frontline worker’ who has to physically travel to work during lockdown?  Examples include people employed in health and social care, education and childcare, local/national government, food production or sale, the prison service, the police and public transport. | • Yes  • No | | | | | | | | | | | |
| What is the highest level of education that you have completed? | • Primary school (0)  • Secondary school up to 16 years (1)  • Higher or secondary or further education (A-levels, BTEC, etc.) (2)  • College or university (3)  • Post-graduate degree (4) | | | | | | | | | | | |
| In the last month, was your household income sufficient to cover the basic needs of your household, such as food and heating? | • Yes  • Mostly  • Sometimes  • No | | | | | | | | | | | |
| Please select the box that best describes your current housing situation: | • I own my home outright  • I own my home and I am paying a mortgage  • I am renting privately  • I am renting from the council/housing association  • I am staying with friends or family  • I am homeless or living in temporary accommodation  • Other | | | | | | | | | | | |
| Do you currently claim Universal Credit? | • Yes, I have applied to receive Universal Credit but have **not yet** received any payments  • Yes, I have claimed Universal Credit and received **one or more** payments  • No | | | | | | | | | | | |
| How many bedrooms are there in your current accommodation? | • 1–10+ | | | | | | | | | | | |
| Do you live alone? | • Yes  • No | | | | | | | | | | | |
| How many people other than yourself live in your household? | • Children aged 0–4 years  ▪ 0–10+  • Children aged 5–15 years  ▪ 0–10+  • People aged 16–64 years  ▪ 0–10+  • People aged 65 years or more  ▪ 0–10+ | | | | | | | | | | | |
| Does your household have any pets? | • Yes  • No | | | | | | | | | | | |
| Which of the following best describes your current occupational status? | • Employed  • Self-employed  • Retired  • Furloughed  • Unemployed  • Student  • Other | | | | | | | | | | | |
| Please indicate which types of pet you have at home.  Select all that apply | • Cat  • Dog  • Indoor bird (e.g. budgie, parrot, canary)  • Rabbit / Guinea pig / Hamster  • Tortoise, turtle, lizard or snake  • Other | | | | | | | | | | | |
| **Behavioural** | | | | | | | | | | | | |
| In the last week, and on average, how many times per day did you wash your hands with soap and water or use hand sanitiser gel? | • 1–10+ | | | | | | | | | | | |
| In the last week, how frequently have you worn a face mask while in an indoor public space? | • Always (100% of the time)  • Usually (50–95% of the time)  • Sometimes (1–49% of the time)  • Never (0% of the time)  • Not applicable (I haven’t been in a public space in the last week) | | | | | | | | | | | |
| In the last week, how frequently have you worn gloves as an infection protection measure while outside the home? | • Always (100% of the time)  • Usually (50–95% of the time)  • Sometimes (1–49% of the time)  • Never (0% of the time)  • Not applicable (I haven’t been in a public space in the last week) | | | | | | | | | | | |
| Over the last week, how many days did you work/study exclusively from home? | • 0 | • 1 | | • 2 | • 3 | • 4 | | • 5 | • 6 | • 7 |  | |
|  | • NA  • I am not currently working or studying | | | | | | | | | | | |
| In the last week, how many journeys did you make on public transport? (return trips count as 2 journeys) | • 1–10+ | | | | | | | | | | | |
| In the last week, how many times have you been inside a shop or supermarket? | • 1–10+ | | | | | | | | | | | |
| In the last week, how often have you been inside another indoor public space (e.g. café, pub, place of worship, restaurant, gym, day centre, waiting room, school, library, entertainment venue, hairdresser, takeaway restaurant)? | • 1–10+ | | | | | | | | | | | |
| Did you travel outside of the UK between 1st November 2019 and 31st October 2020? | • Yes  • No | | | | | | | | | | | |
| Have you travelled outside of the UK since February 2020? | • Yes  • No | | | | | | | | | | | |
| Over the last week, how many times have you been into the home of someone who does not live in your household?  (We are referring to visits in which you enter inside someone else’s home. Visits where you do not cross the threshold do not count. Neither do visits to the shops or other public places, we will ask about these later.) | • 1–10+ | | | | | | | | | | | |
| Have you been advised by a doctor or other professional that you should be ‘SHIELDED’ during the coronavirus outbreak?  (‘Shielding’ involves staying at home and minimising face-to-face contact with people outside the home. This advice has been given to people with certain underlying conditions that place them at increased risk of severe illness from COVID-19, including solid organ transplant recipients; people with specific cancers; people with severe respiratory conditions; people with severe combined immunodeficiency (SCID) or homozygous sickle cell disease; people on certain immunosuppressive treatments; and pregnant women with significant heart disease.) | • Yes  • No | | | | | | | | | | | |
| **Comorbidities** | | | | | | | | | | | | |
| What is your **current** height? (if you are unsure, please put your best estimate) | • Feet/inches  • Centimetres | | | | | | | | | | | |
| What is your **current** weight? | • Stones (sts) / pounds (lbs)  • Kilograms (kg) | | | | | | | | | | | |
| Have you ever been diagnosed with any of the following conditions by a doctor?  Select all that apply. | • Asthma  • Atopic Eczema or Atopic Dermatitis  • Autoimmune disease (e.g. rheumatoid arthritis, multiple sclerosis (MS), lupus (SLE), Crohn’s disease, ulcerative colitis, psoriasis, Raynaud’s disease, scleroderma)  • Cancer  • Cerebral Palsy  • COPD (including chronic bronchitis, and emphysema)  • Cystic Fibrosis  • Dementia  • Diabetes or pre-diabetes  • Hayfever or Allergic Rhinitis  • Heart Attack, Angina or Coronary Artery Disease  • Heart Failure  • High Blood Pressure (Hypertension)  • HIV Infection  • Hyperparathyroidism (overactive parathyroid gland)  • Kidney stones  • Other kidney disease  • Leg Artery Disease (also known as ‘peripheral vascular disease’, ‘peripheral arterial disease’ or ‘intermittent claudication’)  • Mental health disorder  • Motor Neurone Disease  • Organ transplant  • Parkinson's Disease  • Primary immune deficiency (e.g. antibody deficiency, combined immunodeficiency)  • Sarcoidosis  • Sickle Cell Disease (i.e. two copies of altered gene, affected by anaemia and other complications  • Sickle Cell Carrier (also known as ‘sickle cell trait’, with only one copy of altered gene: few symptoms if any)  • Splenectomy (removal of spleen)  • Stroke or Mini-Stroke  • Tuberculosis (TB)  • None of the above | | | | | | | | | | | |
| You indicated you have been diagnosed with diabetes or pre-diabetes. Please specify your diagnosis: | • Pre-diabetes (high blood sugar levels, not enough to be diagnosed with diabetes)  • Type 1 diabetes  • Type 2 diabetes  • Other type of diabetes | | | | | | | | | | | |
| Do you currently have cancer? | • Never  • No, cancer cured or in remission  • Yes, currently receiving treatment | | | | | | | | | | | |
| **[The following three questions on periodontitis were added to the baseline questionnaire from May 20, 2021; participants who were already enrolled were sent the question within the May 20, 2021, monthly follow-up questionnaire]** | | | | | | | | | | | | |
| Gum disease (periodontal disease, periodontitis) is a common problem with the mouth. People with gum disease might have bleeding gums around the teeth, swollen gums, receding gums, or sore or infected gums with symptoms lasting for at least 2 weeks and not caused by an injury or problems with dentures.  **Do you think you might have gum disease?** | • Yes  • No | | | | | | | | | | | |
| Have you ever had any adult teeth that became loose on their own, without some injury? (Milk teeth / baby teeth don’t count) | • Yes  • No | | | | | | | | | | | |
| Have you ever been told by a dental professional that you have lost bone around your teeth? | • Yes  • No | | | | | | | | | | | |
| Under each heading, please click the ONE box that best describes your health TODAY.  (Anxiety / Depression) | • I am not anxious or depressed  • I am moderately anxious or depressed  • I am extremely anxious or depressed | | | | | | | | | | | |
| Over the last 12 months, would you say that on the whole, your health has been: | • Excellent  • Very good  • Good  • Fair  • Poor | | | | | | | | | | | |
| **Vaccination** | | | | | | | | | | | | |
| Have you ever had the BCG vaccine?    *This is the vaccine against Tuberculosis (TB), it's injected in the upper arm and usually leaves a small scar* | • Yes  • No  • Unsure | | | | | | | | | | | |
| Have you ever had the MMR vaccine?  *This is the vaccine against measles, mumps and rubella.* | • Yes  • No  • Unsure | | | | | | | | | | | |
| **Lifestyle** | | | | | | | | | | | | |
| Which of these best describes your use of cigarettes? | • I have never smoked cigarettes  • I used to smoke cigarettes occasionally but now not at all  • I used to smoke cigarettes daily but now not at all  • I smoke cigarettes occasionally but not every day  • I smoke cigarettes daily | | | | | | | | | | | |
| Which of these best describes your use of e‑cigarettes (vaping)? | • I have never vaped or used e-cigarettes  • I used to use e-cigarettes occasionally, but now not at all  • I used to use e-cigarettes daily but now not at all  • I vape occasionally but not every day  • I vape daily | | | | | | | | | | | |
| Are you regularly exposed to smoke from other people’s cigarettes at home or in a car? | • Yes  • No | | | | | | | | | | | |
| Roughly how many hours have you spent outdoors in the last week? | • 0–10+ hours | | | | | | | | | | | |
| During the last week, roughly how many hours did you spend doing more vigorous physical exercise of sufficient intensity to make you breathless or to raise your heart rate significantly, such as heavy physical work, more strenuous gardening (e.g. vigorous digging, landscaping) swimming, jogging, aerobics, football, tennis, cycling, gym workout? | • 0–10+ hours | | | | | | | | | | | |
| During the last week, roughly how many hours did you spend doing lower impact physical exercise to improve flexibility or core strength such as yoga, tai chi or pilates? | • 0–10+ hours | | | | | | | | | | | |
| During the last week, roughly how many hours did you spend doing light exercise that does not make you particularly breathless, such as light gardening, walking, including walking for pleasure or exercise, walking to the shops, walking to work? | • 0–10+ hours | | | | | | | | | | | |
| During the past month, how many hours of actual sleep did you get per night on average?  (This may be different than the number of hours you spent in bed) | • 0–24 hours | | | | | | | | | | | |
| **Diet** | | | | | | | | | | | | |
| Do you **exclude** any of the following foods from your diet?  Select all that apply. | • Eggs  • Cow’s milk of products made from cow’s milk (e.g. cheese, yoghurt)  • Fish  • White meat (e.g. poultry)  • Red meat  • No, I eat all of these foods | | | | | | | | | | | |
| Over the **last week**, how many **portions** of the following did you eat **per day**, on average? |  | | | | | | | | | | | |
| Fruit, vegetables and salad?  *1 portion = 80g (e.g. one apple or two broccoli spears or 3 tablespoons of peas or carrots or one bowl of salad)* | • 0–10+ | | | | | | | | | | | |
| Dairy products (e.g. cow’s milk, cheese, yoghurts) or calcium-fortified dairy alternatives (e.g. soya milks, soya yoghurts and soya cheeses)  *1 portion = a cup of milk, a standard pot of yogurt or a piece of cheese about the size of two thumbs together (30g).* | • 0–10+ | | | | | | | | | | | |
| **Oily** fish  e.g. herring, pilchards, salmon, sardines, sprats, trout and mackerel.  *1 portion = a small tin of oily fish (around 100g) or a piece of oily fish about the size of your palm* | • 0–10+ | | | | | | | | | | | |
| **White fish or seafood**  e.g. cod, haddock, plaice, prawns and tuna  *1 portion = a small (160g) tin of tuna or a piece of white fish about the size of your palm (140g) or 100g prawns* | • 0–10+ | | | | | | | | | | | |
| Over the last week, how many cups or glasses of fluid did you drink per day, on average?  *1 cup or glass = about 150ml. Non-alcoholic drinks including water, tea, coffee, milk and other soft drinks all count* | • 0–10+ | | | | | | | | | | | |
| How many units of alcohol did you drink over the last 7 days?  *One unit is ½ a pint (285 ml) of ordinary beer, lager or cider; 25ml of spirits; 1 small glass (75ml) of wine; or 50ml of sherry* | • None  • 1–7 units  • 8–14 units  • 15–21 units  • 22–28 units  • More than 28 units | | | | | | | | | | | |
| Over the **last month**, have you taken any of the following supplements at least **once per week**?  Select all that apply. | • Multivitamin (including prenatal multivitamins)  • Supplement containing vitamin A only  • Supplement containing vitamin B only  • Supplement containing vitamin C only  • Supplement containing vitamin D only  • Supplement containing calcium only  • Supplement containing calcium and vitamin D combined  • Supplement containing vitamin E only  • Supplement containing zinc only  • Supplement containing iron only  • Supplement containing probiotics  • Supplement containing fish oil, krill oil or other source of omega-3 fatty acids  • Supplement containing cod liver oil  • Supplement containing echinacea  • Supplement containing garlic or garlic powder (allicin)  • Supplement containing turmeric / curcumin  • Supplement containing Cannabidiol (CBD) oil  • Supplements containing folic acid  • Supplement containing Selenium only  • Other (e.g. other micronutrients (such as herbal supplements) or combinations of micronutrients (such vitamin C & zinc)) Please specify:  • None of the above | | | | | | | | | | | |
| **Medications** | | | | | | | | | | | | |
| Please type the names of all the medications you are currently taking below, one medication per box. The next pages will collect details about dosage for each.    Include all types of medications taken at home or administered in a hospital or clinic (capsules, tablets, contraceptive pills or implants, inhalers, injections, intravenous infusions, monoclonal antibodies, chemotherapy, immunosuppressants, etc.)  Please note that other pages will collect details about the amount of medicine in each dose (next page) and how often you take each dose (the page after that). If there are any details about your medication that aren’t captured by our form (e.g. if you take different doses of a medicine at different times of day), there will be space to enter them in a blank text box at the end of this section of the questionnaire. |  | | | | | | | | | | |  |
|  |  | |  | | | | **Medication name (generic or brand name, either is fine)** | | | | |  |
|  |  |  | Medication 1 | | | |  | | | | |  |
|  |  |  | Medication 2 | | | |  | | | | |  |
|  |  |  | …. | | | |  | | | | |  |
|  |  | | | | | | | | | | | |
| Please select the frequency and route that you take your medication: | • Frequency   - 4 times/day - 3 times/day - 2 times/day - One daily - Weekly - Less often than weekly - As needed   • Route   - By mouth - Inhaled - Injected - Other | | | | | | | | | | | |
| **Recent health** | | | | | | | | | | | | |
| Since February 1st 2020, have you experienced any of the following symptoms: loss of smell or taste, fever, persistent cough, fatigue, diarrhoea, abdominal pain or loss of appetite? | • Yes, I have had one or more of these symptoms since 1st of February  • No, I have not had any of these symptoms since 1st of February | | | | | | | | | | | |
| When did your symptoms start?  (DD/MM/YYYY)  *e.g. 25/04/2020* |  | | | | | | | | | | | |
| Did you have a persistent cough (coughing a lot for more than an hour, or 3 or more coughing episodes in 24 hours)? | • No  • Persistent dry cough (i.e. producing little or no phlegm)  • Persistent productive cough | | | | | | | | | | | |
| Did you experience unusual fatigue? | • No  • Mild fatigue  • Severe fatigue – I struggled to get out of bed | | | | | | | | | | | |
| Did you have a loss of sense of smell or taste? | • Yes  • No | | | | | | | | | | | |
| Did you skip any meals because you felt unwell? | • Yes  • No | | | | | | | | | | | |
| Since February 1st 2020, have you had a nose/throat swab to test for COVID-19? | • Yes  • No | | | | | | | | | | | |
| On what date did you have this nose/throat swab?  If you are not sure of the exact date, enter the approximate date (DD/MM/YYYY).  *e.g. 25/04/2020* |  | | | | | | | | | | | |
| What was the result? |  | | | | | | | | | | | |

# ***Table S2:* Monthly follow-up questions**

| **COVID testing** | |
| --- | --- |
| Since you last checked in with us, have you had a nose or throat swab for COVID-19 or any other respiratory virus, or has a result from a previous swab test become newly available?  (This question is about tests to detect the virus itself: they are usually done in somebody who has symptoms, but screening of asymptomatic people can also be done. It’s usually a nose/throat swab, but saliva tests are also becoming available) | • Yes  • No |
| On what date did you have this nose / throat swab?  Only one date should be selected. If you are not sure of the exact date, click on the approximate date.    If you had one or more positive swabs, please select the date of your first positive swab.    If you have had multiple negative swabs, please select the date of your first negative swab. |  |
| What was the result? Click as many as apply. | • Positive for COVID-19 (SARS-CoV-2 coronavirus)  • Positive for influenza virus  • Positive for another respiratory virus  • Negative for all/any viruses tested  • Not known |
| **SARS-CoV-2 vaccinations** |  |
| Since you last checked in with us, have you had one or more doses of a COVID-19 vaccine (immunisation)? First doses and booster doses both count. | • Yes, I have had one or more doses of COVID-19 vaccine since I filled my last questionnaire  • No, I have not had any doses of COVID-19 vaccine since I filled my last questionnaire  • Not sure (e.g. you took part in vaccine trial, but don’t yet know whether or not you had the real vaccine or the placebo (dummy vaccine) |
| On what date did you have this vaccine dose? Please select a date on the calendar below.  If you are not sure of the exact date, click on the approximate date. |  |
| Which COVID-19 vaccine did you have? | • Oxford / AstraZeneca / ChAdOx1  • Pfizer / BioNTech  • Moderna  • Valneva  • Novavax  • Janssen (also known as Johnson & Johnson)  • Other - please specify  • Not sure / don't know |
| **Sociodemographics** |  |
| Since you last checked in with us, have you claimed Universal Credit? | • Yes, I have applied to receive Universal Credit but have not yet received any payments  • Yes, I have claimed Universal Credit and received one or more payments  • No |
| **Lifestyle** |  |
| Which of the following best describes your use of cigarettes over the last month? | • I did not smoke any cigarettes  • I smoked cigarettes occasionally but not everyday  • I smoked cigarettes daily |
| Which of the following best describes you use of e-cigarettes (vaping) over the last month? | • I did not vape or use e-cigarettes  • I smoked vape or used e-cigarettes occasionally but not everyday  • I smoked vape or used e-cigarettes daily |
| During the last week, roughly how many hours did you spend doing light exercise that does not make you particularly breathless, such as light gardening, walking, including walking for pleasure or exercise, walking to the shops, walking to work? | • 0–10+ |
| During the last week, roughly how many hours did you spend doing more vigorous physical exercise of sufficient intensity to make you breathless or to raise your heart rate significantly, such as heavy physical work, more strenuous gardening (e.g. vigorous digging, landscaping) swimming, jogging, aerobics, football, tennis, cycling, gym workout? | • 0–10+ |
| During the last week, roughly how many hours did you spend doing lower impact physical exercise to improve flexibility or core strength such as yoga, tai chi or pilates? | • 0–10+ |
| How many units of alcohol did you drink over the last 7 days?  *One unit is ½ a pint (285 ml) of ordinary beer, lager or cider; 25ml of spirits; 1 small glass (75ml) of wine; or 50ml of sherry* | • None  • 1–7 units  • 8–14 units  • 15–21 units  • 22–28 units  • More than 28 units |
| During the past month, how many hours of actual sleep did you get per night on average? (This may be different than the number of hours you spent in bed) | • 0–24 hours |
| **Diet** |  |
| Over the **last month**, have you taken any of the following supplements at least **once per week**?  Select all that apply. | • Multivitamin (including prenatal multivitamins)  • Supplement containing vitamin A only  • Supplement containing vitamin B only  • Supplement containing vitamin C only  • Supplement containing vitamin D only  • Supplement containing calcium only  • Supplement containing calcium and vitamin D combined  • Supplement containing vitamin E only  • Supplement containing zinc only  • Supplement containing iron only  • Supplement containing probiotics  • Supplement containing fish oil, krill oil or other source of omega-3 fatty acids  • Supplement containing cod liver oil  • Supplement containing echinacea  • Supplement containing garlic or garlic powder (allicin)  • Supplement containing turmeric / curcumin  • Supplement containing Cannabidiol (CBD) oil  • Supplements containing folic acid  • Supplement containing Selenium only  • Other (e.g. other micronutrients (such as herbal supplements) or combinations of micronutrients (such vitamin C & zinc)) Please specify:  • None of the above |
| **Recent health** |  |
| Over the last month, would you say that on the whole, your health has been: | • Excellent  • Very good  • Good  • Fair  • Poor |
| Under each heading, please click the ONE box that best describes your health TODAY.  (Anxiety / Depression) | • I am not anxious or depressed  • I am moderately anxious or depressed  • I am extremely anxious or depressed |
| **Behavioural** |  |
| In the last week, how many journeys did you make on public transport? (return trips count as 2 journeys) | 0–10+ |
| Over the last week, how many times have you been visited at home by someone who does not live in your household?  (We are referring to visitors entering inside your home. People who just call at the door and do not come inside do not count) | 0–10+ |
| Over the last week, how many times have you visited someone who does not live in your house?  (We are referring to visits in which you enter inside someone else’s home. Visits where you do not cross the threshold do not count. Neither do visits to the shops or other public places, we will ask about these later.) | 0–10+ |
| In the last week, how many times have you been inside a shop or supermarket? | 0–10+ |
| In the last week, how often have you been inside another indoor public space (e.g. café, pub, place of worship, restaurant, gym, day centre, waiting room, school, library, entertainment venue, hairdresser, takeaway restaurant)? | 0–10+ |
| Since you last checked in with us, have you travelled outside of the UK? | • Yes  • No |

# ***Figure S2:* Correlation matrix of raw variables: post-primary cohort**

Spearman's ρ is shown. Nominal variables are excluded.

# ***Figure S3:* Correlation matrix of raw variables: post-booster cohort**

Spearman's ρ is shown. Nominal variables are excluded.

# ***Figure S4:* Correlation matrix of the estimators: assessment of multicollinearity for the post-primary analysis**

# ***Figure S5:* Correlation matrix of the estimators: assessment of multicollinearity for the post-booster analysis**

# ***Table S3:* Factors not associated with breakthrough SARS-CoV-2 infection in the post-primary cohort, after adjustment for age and sex**

|  |  | | **HR (95% CI)** | | | **p value** |  | |
| --- | --- | --- | --- | --- | --- | --- | --- | --- |
|  | Ethnicity | |  | | |  |  | |
|  | White | | 1.00 | | |  |  | |
|  | Mixed, multiple, or other ethnic groups | | 1.01 (0.72–1.41) | | | 0.9742 |  | |
|  | South Asian | | 0.75 (0.47–1.18) | | | 0.2099 |  | |
|  | Black, African, Caribbean, or Black British | | 0.42 (0.14–1.31) | | | 0.1340 |  | |
|  | Quartiles of IMD rank | |  | | |  |  | |
|  | Q4 (least deprived) | | 1.00 | | |  |  | |
|  | Q3 | | 0.89 (0.75–1.06) | | | 0.1903 |  | |
|  | Q2 | | 1.05 (0.89–1.25) | | | 0.5369 |  | |
|  | Q1 (most deprived) | | 0.90 (0.76–1.07) | | | 0.2333 |  | |
|  | Claiming Universal Credit | |  | | |  |  | |
|  | No | | 1.00 | | |  |  | |
|  | Yes | | 1.10 (0.78–1.54) | | | 0.5883 |  | |
|  | Dog at home | |  | | |  |  | |
|  | No | | 1.00 | | |  |  | |
|  | Yes | | 0.94 (0.81–1.08) | | | 0.3802 |  | |
|  | Smoking status | |  | | |  |  | |
|  | No | | 1.00 | | |  |  | |
|  | Yes | | 1.12 (0.87–1.45) | | | 0.3863 |  | |
|  | Environmental tobacco smoke exposure | |  | | |  |  | |
|  | No | | 1.00 | | |  |  | |
|  | Yes | | 1.03 (0.68–1.58) | | | 0.8813 |  | |
|  | Travel outside of the UK since last questionnaire | |  | | |  |  | |
|  | No | | 1.00 | | |  |  | |
|  | Yes | | 1.07 (0.91–1.25) | | | 0.4161 |  | |
|  | Weekly journeys on public transport | |  | | |  |  | |
|  | 0 | | 1.00 | | |  |  | |
|  | 1–5 | | 1.01 (0.87–1.17) | | | 0.9186 |  | |
|  | ≥6 | | 1.01 (0.81–1.26) | | | 0.9126 |  | |
|  | Lower impact physical exercise, h per week | |  | | |  |  | |
|  | 0 | | 0.97 (0.84–1.13) | | | 0.7236 |  | |
|  | 1 | | 0.92 (0.76–1.12) | | | 0.4224 |  | |
|  | ≥2 | | 1.00 | | |  |  | |
|  | Light physical exercise, h per week | |  | | |  |  | |
|  | 0–2 | | 1.01 (0.84–1.21) | | | 0.8984 |  | |
|  | 3–5 | | 1.13 (0.95–1.34) | | | 0.1568 |  | |
|  | 7–9 | | 1.06 (0.88–1.28) | | | 0.5331 |  | |
|  | ≥10 | | 1.00 | | |  |  | |
|  | Vigorous physical exercise,  h per week | |  | | |  |  | |
|  | 0 | | 0.95 (0.80–1.13) | | | 0.5615 |  | |
|  | 1–3 | | 0.99 (0.84–1.18) | | | 0.9235 |  | |
|  | ≥4 | | 1.00 | | |  |  | |
|  | Actual sleep, h/night | |  | | |  |  | |
|  | ≤5 | | 0.96 (0.78–1.19) | | | 0.7239 |  | |
|  | 6 | | 1.09 (0.94–1.27) | | | 0.2590 |  | |
|  | 7 | | 1.00 | | |  |  | |
|  | ≥8 | | 0.91 (0.77–1.06) | | | 0.2208 |  | |
|  | Multivitamin supplements | |  | | |  |  | |
|  | No | | 1.00 | | |  |  | |
|  | Yes | | 1.08 (0.94–1.24) | | | 0.2866 |  | |
|  | Vitamin A supplements | |  | | |  |  | |
|  | No | | 1.00 | | |  |  | |
|  | Yes | | 0.92 (0.48–1.77) | | | 0.7979 |  | |
|  | Vitamin C supplements | |  | | |  |  | |
|  | No | | 1.00 | | |  |  | |
|  | Yes | | 1.06 (0.88–1.28) | | | 0.5165 |  | |
|  |  | |  | | |  |  | |
|  |  | | **HR (95% CI)** | | | **p value** |  | |
|  | Vitamin D supplements | |  | | |  |  | |
|  | No | | 1.00 | | |  |  | |
|  | Yes | | 1.02 (0.90–1.15) | | | 0.7521 |  | |
|  | Zinc supplements | |  | | |  |  | |
|  | No | | 1.00 | | |  |  | |
|  | Yes | | 1.05 (0.83–1.32) | | | 0.6946 |  | |
|  | Selenium supplements | |  | | |  |  | |
|  | No | | 1.00 | | |  |  | |
|  | Yes | | 1.07 (0.63–1.82) | | | 0.7900 |  | |
|  | Iron supplements | |  | | |  |  | |
|  | No | | 1.00 | | |  |  | |
|  | Yes | | 0.77 (0.55–1.09) | | | 0.1379 |  | |
|  | Probiotics | |  | | |  |  | |
|  | No | | 1.00 | | |  |  | |
|  | Yes | | 0.97 (0.75–1.25) | | | 0.7923 |  | |
|  | Fish oil, krill oil, or other omega-3 supplements | |  | | |  |  | |
|  | No | | 1.00 | | |  |  | |
|  | Yes | | 0.98 (0.80–1.19) | | | 0.8024 |  | |
|  | Cod liver oil supplements | |  | | |  |  | |
|  | No | | 1.00 | | |  |  | |
|  | Yes | | 1.02 (0.80–1.28) | | | 0.8992 |  | |
|  | Garlic or allicin supplements | |  | | |  |  | |
|  | No | | 1.00 | | |  |  | |
|  | Yes | | 0.88 (0.51–1.51) | | | 0.6336 |  | |
|  | Daily portions of dairy products or calcium-fortified alternatives | |  | | |  |  | |
|  | 0 | | 1.00 | | |  |  | |
|  | 1 | | 0.95 (0.68–1.32) | | | 0.7583 |  | |
|  | 2–3 | | 1.01 (0.73–1.38) | | | 0.9610 |  | |
|  | ≥4 | | 1.00 (0.72–1.38) | | | 0.9932 |  | |
|  | Food choice | |  | | |  |  | |
|  | None | | 1.00 | | |  |  | |
|  | Vegetarian | | 0.98 (0.74–1.29) | | | 0.8779 |  | |
|  | Vegan | | 1.16 (0.75–1.79) | | | 0.4991 |  | |
|  | BMI, kg/m² | |  | | |  |  | |
|  | <25 | | 1.00 | | |  |  | |
|  | 25 to <30 | | 1.02 (0.88–1.17) | | | 0.8154 |  | |
|  | ≥30 | | 1.11 (0.95–1.30) | | | 0.1742 |  | |
|  | Heart disease | |  | | |  |  | |
|  | No | | 1.00 | | |  |  | |
|  | Yes | | 0.96 (0.66–1.40) | | | 0.8513 |  | |
|  | Arterial disease | |  | | |  |  | |
|  | No | | 1.00 | | |  |  | |
|  | Yes | | 0.92 (0.66–1.28) | | | 0.6178 |  | |
|  | Periodontitis | |  | | |  |  | |
|  | No | | 1.00 | | |  |  | |
|  | Yes | | 1.03 (0.90–1.19) | | | 0.6583 |  | |
|  | Major neurological conditions | |  | | |  |  | |
|  | No | | 1.00 | | |  |  | |
|  | Yes | | 1.10 (0.74–1.62) | | | 0.6484 |  | |
|  | Asthma | |  | | |  |  | |
|  | No | | 1.00 | | |  |  | |
|  | Yes | | 1.02 (0.87–1.19) | | | 0.8178 |  | |
|  | COPD | |  | | |  |  | |
|  | No | | 1.00 | | |  |  | |
|  | Yes | | 0.74 (0.42–1.27) | | | 0.2723 |  | |
|  | Atopy | |  | | |  |  | |
|  | No | | 1.00 | | |  |  | |
|  | Yes | | 0.96 (0.84–1.10) | | | 0.5456 |  | |
|  | Cancer | |  | | |  |  | |
|  | Never | | 1.00 | | |  |  | |
|  | Past (cured or in remission) | | 0.92 (0.72–1.18) | | | 0.5302 |  | |
|  | Present (active treatment) | | 1.10 (0.55–2.22) | | | 0.7808 |  | |
|  |  | |  | | |  |  | |
|  | |  | | **HR (95% CI)** | **p value** | |  |  |
|  | Diabetes type | |  | | |  |  |  |
|  | No diabetes | | 1.00 | | |  |  |  |
|  | Pre-diabetes | | 0.94 (0.63–1.40) | | | 0.7481 |  |  |
|  | Type 1 diabetes | | 0.74 (0.33–1.66) | | | 0.4656 |  |  |
|  | Type 2 diabetes | | 0.96 (0.69–1.34) | | | 0.8088 |  |  |
|  | | Beta-2 adrenergic agonists | |  |  | |  |  |
|  | | No | | 1.00 |  | |  |  |
|  | | Yes | | 0.87 (0.71–1.07) | 0.1925 | |  |  |
|  | | Beta blockers | |  |  | |  |  |
|  | | No | | 1.00 |  | |  |  |
|  | | Yes | | 1.13 (0.88–1.45) | 0.3541 | |  |  |
|  | | Statins | |  |  | |  |  |
|  | | No | | 1.00 |  | |  |  |
|  | | Yes | | 0.98 (0.80–1.19) | 0.8140 | |  |  |
|  | | ACE inhibitors | |  |  | |  |  |
|  | | No | | 1.00 |  | |  |  |
|  | | Yes | | 0.84 (0.66–1.07) | 0.1593 | |  |  |
|  | | Proton pump inhibitors | |  |  | |  |  |
|  | | No | | 1.00 |  | |  |  |
|  | | Yes | | 0.98 (0.81–1.18) | 0.8192 | |  |  |
|  | | H2-receptor antagonists | |  |  | |  |  |
|  | | No | | 1.00 |  | |  |  |
|  | | Yes | | 0.77 (0.32–1.84) | 0.5512 | |  |  |
|  | | Inhaled corticosteroids | |  |  | |  |  |
|  | | No | | 1.00 |  | |  |  |
|  | | Yes | | 0.89 (0.69–1.15) | 0.3720 | |  |  |
|  | | Bronchodilators | |  |  | |  |  |
|  | | No | | 1.00 |  | |  |  |
|  | | Yes | | 0.88 (0.72–1.08) | 0.2312 | |  |  |
|  | | Systemic immunosuppressants | |  |  | |  |  |
|  | | No | | 1.00 |  | |  |  |
|  | | Yes | | 0.99 (0.74–1.31) | 0.9215 | |  |  |
|  | | Angiotensin receptor blockers | |  |  | |  |  |
|  | | No | | 1.00 |  | |  |  |
|  | | Yes | | 1.03 (0.77–1.36) | 0.8606 | |  |  |
|  | | SSRIs | |  |  | |  |  |
|  | | No | | 1.00 |  | |  |  |
|  | | Yes | | 0.96 (0.76–1.20) | 0.6929 | |  |  |
|  | | Non-SSRI antidepressants | |  |  | |  |  |
|  | | No | | 1.00 |  | |  |  |
|  | | Yes | | 0.83 (0.60–1.15) | 0.2688 | |  |  |
|  | | Thiazides | |  |  | |  |  |
|  | | No | | 1.00 |  | |  |  |
|  | | Yes | | 0.78 (0.50–1.22) | 0.2848 | |  |  |
|  | | Vitamin K antagonists | |  |  | |  |  |
|  | | No | | 1.00 |  | |  |  |
|  | | Yes | | 0.79 (0.33–1.91) | 0.6023 | |  |  |
|  | | SGLT2 inhibitors | |  |  | |  |  |
|  | | No | | 1.00 |  | |  |  |
|  | | Yes | | 1.70 (0.85–3.42) | 0.1351 | |  |  |
|  | | Metformin | |  |  | |  |  |
|  | | No | | 1.00 |  | |  |  |
|  | | Yes | | 1.17 (0.81–1.69) | 0.3976 | |  |  |
|  | | Bisphosphonates | |  |  | |  |  |
|  | | No | | 1.00 |  | |  |  |
|  | | Yes | | 0.70 (0.38–1.31) | 0.2681 | |  |  |
|  | | Anti-platelet drugs | |  |  | |  |  |
|  | | No | | 1.00 |  | |  |  |
|  | | Yes | | 1.05 (0.80–1.39) | 0.7064 | |  |  |
|  | | Sex hormone therapy | |  |  | |  |  |
|  | | No | | 1.00 |  | |  |  |
|  | | Yes | | 1.01 (0.81–1.26) | 0.9154 | |  |  |
|  | | Aspirin | |  |  | |  |  |
|  | | No | | 1.00 |  | |  |  |
|  | | Yes | | 1.14 (0.85–1.54) | 0.3843 | |  |  |
|  | |  | |  |  | |  |  |
|  | |  | | **HR (95% CI)** | **p value** | |  |  |
|  | | Paracetamol | |  |  | |  |  |
|  | | No | | 1.00 |  | |  |  |
|  | | Yes | | 1.14 (0.85–1.52) | 0.3780 | |  |  |
|  | | BCG vaccinated | |  |  | |  |  |
|  | | No | | 1.00 |  | |  |  |
|  | | Yes | | 0.99 (0.83–1.19) | 0.9448 | |  |  |
|  | |  | |  |  | |  |  |

ACE = angiotensin-converting-enzyme. BCG = Bacille Calmette Guérin. BMI = body-mass index. COPD = chronic obstructive pulmonary disease. HR = hazard ratio. IMD = Index of Multiple Deprivation. SLT2 = sodium-glucose co-transporter-2. SSRI = selective serotonin reuptake inhibitors.

# ***Table S4:* Factors not associated with breakthrough SARS-CoV-2 infection in the post-booster cohort, after adjustment for age and sex**

|  |  | **HR (95% CI)** | **p value** |  |
| --- | --- | --- | --- | --- |
|  | Number of people per bedroom |  |  |  |
|  | <1 | 1.00 |  |  |
|  | 1 to <2 | 1.09 (0.94–1.26) | 0.2673 |  |
|  | ≥2 | 1.36 (0.89–2.10) | 0.1579 |  |
|  | Quartiles of IMD rank |  |  |  |
|  | Q4 (least deprived) | 1.00 |  |  |
|  | Q3 | 0.91 (0.77–1.08) | 0.3048 |  |
|  | Q2 | 1.00 (0.84–1.19) | 0.9882 |  |
|  | Q1 (most deprived) | 0.99 (0.83–1.18) | 0.8787 |  |
|  | Claiming Universal Credit |  |  |  |
|  | No | 1.00 |  |  |
|  | Yes | 0.94 (0.59–1.50) | 0.7891 |  |
|  | Dog at home |  |  |  |
|  | No | 1.00 |  |  |
|  | Yes | 1.08 (0.93–1.25) | 0.3134 |  |
|  | Smoking status |  |  |  |
|  | No | 1.00 |  |  |
|  | Yes | 0.93 (0.67–1.30) | 0.6638 |  |
|  | Vaping status |  |  |  |
|  | No | 1.00 |  |  |
|  | Yes | 1.10 (0.75–1.60) | 0.6271 |  |
|  | Environmental tobacco smoke exposure |  |  |  |
|  | No | 1.00 |  |  |
|  | Yes | 1.15 (0.73–1.81) | 0.5421 |  |
|  | Lower impact physical exercise, h per week |  |  |  |
|  | 0 | 1.05 (0.89–1.24) | 0.5468 |  |
|  | 1 | 1.13 (0.93–1.37) | 0.2314 |  |
|  | ≥2 | 1.00 |  |  |
|  | Light physical exercise,  h per week |  |  |  |
|  | 0–2 | 0.91 (0.76–1.10) | 0.3404 |  |
|  | 3–5 | 1.01 (0.86–1.18) | 0.9084 |  |
|  | 7–9 | 0.92 (0.77–1.09) | 0.3409 |  |
|  | ≥10 | 1.00 |  |  |
|  | Multivitamin supplements |  |  |  |
|  | No | 1.00 |  |  |
|  | Yes | 0.89 (0.76–1.04) | 0.1486 |  |
|  | Vitamin A supplements |  |  |  |
|  | No | 1.00 |  |  |
|  | Yes | 0.96 (0.43–2.13) | 0.9116 |  |
|  | Vitamin C supplements |  |  |  |
|  | No | 1.00 |  |  |
|  | Yes | 1.00 (0.81–1.24) | 0.9857 |  |
|  | Vitamin D supplements |  |  |  |
|  | No | 1.00 |  |  |
|  | Yes | 0.99 (0.88–1.12) | 0.9008 |  |
|  | Zinc supplements |  |  |  |
|  | No | 1.00 |  |  |
|  | Yes | 0.86 (0.65–1.13) | 0.2849 |  |
|  | Selenium supplements |  |  |  |
|  | No | 1.00 |  |  |
|  | Yes | 0.80 (0.40–1.61) | 0.5362 |  |
|  | Iron supplements |  |  |  |
|  | No | 1.00 |  |  |
|  | Yes | 0.82 (0.56–1.21) | 0.3167 |  |
|  | Garlic or allicin supplements |  |  |  |
|  | No | 1.00 |  |  |
|  | Yes | 0.87 (0.50–1.50) | 0.6163 |  |
|  |  |  |  |  |

|  | |  | | | **HR (95% CI)** | | **p value** | |  | |
| --- | --- | --- | --- | --- | --- | --- | --- | --- | --- | --- |
|  | | Daily portions of fruit, vegetable, and salad | |  | | |  | |  | |
|  | | 0–2 | | 1.00 | | |  | |  | |
|  | | 3–4 | | 1.00 (0.82–1.22) | | | 0.9907 | |  | |
|  | | 5 | | 0.90 (0.72–1.13) | | | 0.3732 | |  | |
|  | | ≥6 | | 0.96 (0.78–1.18) | | | 0.7044 | |  | |
|  | | Daily portions of dairy products or calcium-fortified alternatives | | |  | |  | |  | |
|  | | 0 | | | 1.29 (0.92–1.80) | | 0.1395 | |  | |
|  | | 1 | | | 0.97 (0.82–1.16) | | 0.7647 | |  | |
|  | | 2–3 | | | 0.97 (0.83–1.12) | | 0.6730 | |  | |
|  | | ≥4 | | | 1.00 | |  | |  | |
|  | | Food choice | | |  | |  | |  | |
|  | | None | | | 1.00 | |  | |  | |
|  | | Vegetarian | | | 0.81 (0.59–1.12) | | 0.2017 | |  | |
|  | | Vegan | | | 0.79 (0.45–1.40) | | 0.4197 | |  | |
|  | | General health | | |  | |  | |  | |
|  | | Excellent | | | 1.00 | |  | |  | |
|  | | Very good | | | 1.04 (0.86–1.25) | | 0.7048 | |  | |
|  | | Good | | | 0.99 (0.81–1.21) | | 0.9235 | |  | |
|  | | Fair | | | 0.82 (0.64–1.05) | | 0.1138 | |  | |
|  | | Poor | | | 0.90 (0.61–1.33) | | 0.6075 | |  | |
|  | | BMI, kg/m² | | |  | |  | |  | |
|  | | <25 | | | 1.00 | |  | |  | |
|  | | 25 to <30 | | | 1.08 (0.93–1.24) | | 0.3118 | |  | |
|  | | ≥30 | | | 0.98 (0.82–1.16) | | 0.7769 | |  | |
|  | | Heart disease | | |  | |  | |  | |
|  | | No | | | 1.00 | |  | |  | |
|  | | Yes | | | 0.92 (0.65–1.32) | | 0.6648 | |  | |
|  | | Arterial disease | | |  | |  | |  | |
|  | | No | | | 1.00 | |  | |  | |
|  | | Yes | | | 0.89 (0.65–1.21) | | 0.4588 | |  | |
|  | | Periodontitis | | |  | |  | |  | |
|  | | No | | | 1.00 | |  | |  | |
|  | | Yes | | | 1.01 (0.88–1.16) | | 0.8682 | |  | |
|  | | Hypertension | | |  | |  | |  | |
|  | | No | | | 1.00 | |  | |  | |
|  | | Yes | | | 0.88 (0.75–1.04) | | 0.1390 | |  | |
|  | | Immunodeficiency | | |  | |  | |  | |
|  | | No | | | 1.00 | |  | |  | |
|  | | Yes | | | 1.22 (0.67–2.21) | | 0.5145 | |  | |
|  | | Major neurological conditions | | |  | |  | |  | |
|  | | No | | | 1.00 | |  | |  | |
|  | | Yes | | | 0.72 (0.46–1.13) | | 0.1570 | |  | |
|  | | Asthma | | |  | |  | |  | |
|  | | No | | | 1.00 | |  | |  | |
|  | | Yes | | | 0.99 (0.84–1.16) | | 0.8976 | |  | |
|  | | COPD | | |  | |  | |  | |
|  | | No | | | 1.00 | |  | |  | |
|  | | Yes | | | 0.89 (0.55–1.41) | | 0.6107 | |  | |
|  | | Cancer | | |  | |  | |  | |
|  | | Never | | | 1.00 | |  | |  | |
|  | | Past (cured or in remission) | | | 0.88 (0.69–1.10) | | 0.2636 | |  | |
|  | | Present (active treatment) | | | 0.88 (0.44–1.77) | | 0.7230 | |  | |
|  | | Beta-2 adrenergic agonists | | |  | |  | |  | |
|  | | No | | | 1.00 | |  | |  | |
|  | | Yes | | | 0.87 (0.70–1.08) | | 0.2063 | |  | |
|  | | Statins | | |  | |  | |  | |
|  | | No | | | 1.00 | |  | |  | |
|  | | Yes | | | 0.95 (0.79–1.13) | | 0.5465 | |  | |
|  | | Proton pump inhibitors | | |  | |  | |  | |
|  | | No | | | 1.00 | |  | |  | |
|  | | Yes | | | 0.86 (0.72–1.04) | | 0.1183 | |  | |
|  | |  | | |  | |  | |  | |
|  |  | | **HR (95% CI)** | | | **p value** | |  | |  |
|  | H2-receptor antagonists | |  | | |  | |  | |  |
|  | No | | 1.00 | | |  | |  | |  |
|  | Yes | | 0.88 (0.40–1.97) | | | 0.7605 | |  | |  |
|  | Inhaled corticosteroids | |  | | |  | |  | |  |
|  | No | | 1.00 | | |  | |  | |  |
|  | Yes | | 0.97 (0.76–1.24) | | | 0.8216 | |  | |  |
|  | Bronchodilators | |  | | |  | |  | |  |
|  | No | | 1.00 | | |  | |  | |  |
|  | Yes | | 0.89 (0.72–1.10) | | | 0.2723 | |  | |  |
|  | Systemic immunosuppressants | |  | | |  | |  | |  |
|  | No | | 1.00 | | |  | |  | |  |
|  | Yes | | 0.89 (0.68–1.17) | | | 0.4038 | |  | |  |
|  | SSRIs | |  | | |  | |  | |  |
|  | No | | 1.00 | | |  | |  | |  |
|  | Yes | | 0.83 (0.63–1.08) | | | 0.1695 | |  | |  |
|  | Non-SSRI antidepressants | |  | | |  | |  | |  |
|  | No | | 1.00 | | |  | |  | |  |
|  | Yes | | 0.89 (0.65–1.22) | | | 0.4770 | |  | |  |
|  | Calcium channel blockers | |  | | |  | |  | |  |
|  | No | | 1.00 | | |  | |  | |  |
|  | Yes | | 0.96 (0.77–1.20) | | | 0.7485 | |  | |  |
|  | Vitamin K antagonists | |  | | |  | |  | |  |
|  | No | | 1.00 | | |  | |  | |  |
|  | Yes | | 0.92 (0.43–1.93) | | | 0.8155 | |  | |  |
|  | SGLT2 inhibitors | |  | | |  | |  | |  |
|  | No | | 1.00 | | |  | |  | |  |
|  | Yes | | 0.21 (0.03–1.47) | | | 0.1152 | |  | |  |
|  | Anticholinergics | |  | | |  | |  | |  |
|  | No | | 1.00 | | |  | |  | |  |
|  | Yes | | 0.82 (0.61–1.09) | | | 0.1723 | |  | |  |
|  |  | | **HR (95% CI)** | | | **p value** | |  | |  |
|  | Bisphosphonates | |  | | |  | |  | |  |
|  | No | | 1.00 | | |  | |  | |  |
|  | Yes | | 0.68 (0.40–1.16) | | | 0.1564 | |  | |  |
|  | Anti-platelet drugs | |  | | |  | |  | |  |
|  | No | | 1.00 | | |  | |  | |  |
|  | Yes | | 0.94 (0.72–1.22) | | | 0.6252 | |  | |  |
|  | Sex hormone therapy | |  | | |  | |  | |  |
|  | No | | 1.00 | | |  | |  | |  |
|  | Yes | | 1.12 (0.90–1.39) | | | 0.2975 | |  | |  |
|  | Aspirin | |  | | |  | |  | |  |
|  | No | | 1.00 | | |  | |  | |  |
|  | Yes | | 0.91 (0.68–1.23) | | | 0.5548 | |  | |  |
|  | Paracetamol | |  | | |  | |  | |  |
|  | No | | 1.00 | | |  | |  | |  |
|  | Yes | | 0.83 (0.60–1.15) | | | 0.2619 | |  | |  |
|  | BCG vaccinated | |  | | |  | |  | |  |
|  | No | | 1.00 | | |  | |  | |  |
|  | Yes | | 1.08 (0.89–1.32) | | | 0.4448 | |  | |  |
|  |  | |  | | |  | |  | |  |

ACE = angiotensin-converting-enzyme. BCG = Bacille Calmette Guérin. BMI = body-mass index. COPD = chronic obstructive pulmonary disease. HR = hazard ratio. IMD = Index of Multiple Deprivation. SLT2 = sodium-glucose co-transporter-2. SSRI = Selective serotonin reuptake inhibitors.

# ***Table S5:* Trend analysis showing tests for linear, quadratic, cubic, and quartic trends of ordinal variables**

|  |  | **p for trend** | |  |
| --- | --- | --- | --- | --- |
|  |  | Post-primary | Post-booster |  |
|  | Highest education level attained |  |  |  |
|  | Linear | <0.0001 | 0.0003 |  |
|  | Quadratic | 0.1793 | 0.6650 |  |
|  | Cubic | 0.2608 | 0.6192 |  |
|  | Number of people per bedroom |  |  |  |
|  | Linear | 0.0122 | .. |  |
|  | Quadratic | 0.1818 | .. |  |
|  | Multigenerational households |  |  |  |
|  | Linear | 0.2420 | 0.5742 |  |
|  | Quadratic | 0.6136 | 0.4039 |  |
|  | Alcohol consumption, units per week |  |  |  |
|  | Linear | 0.0667 | 0.0584 |  |
|  | Quadratic | 0.9008 | 0.1732 |  |
|  | Cubic | 0.7115 | 0.1528 |  |
|  | Weekly journeys on public transport |  |  |  |
|  | Linear | .. | 0.4105 |  |
|  | Quadratic | .. | 0.3978 |  |
|  | Weekly visits to shops |  |  |  |
|  | Linear | 0.4450 | 0.0258 |  |
|  | Quadratic | 0.4282 | 0.3285 |  |
|  | Cubic | 0.4389 | 0.4936 |  |
|  | Weekly visits to other indoor public places (not shops) |  |  |  |
|  | Linear | 0.0011 | 0.0049 |  |
|  | Quadratic | 0.9200 | 0.4764 |  |
|  | Vigorous physical exercise, h per week |  |  |  |
|  | Linear | .. | 0.2250 |  |
|  | Quadratic | .. | 0.4022 |  |
|  | Actual sleep, h/night |  |  |  |
|  | Linear | .. | 0.0555 |  |
|  | Quadratic | .. | 0.1209 |  |
|  | Cubic | .. | 0.1850 |  |
|  | Daily portions of fruit, vegetables, and salad |  |  |  |
|  | Linear | 0.1598 | .. |  |
|  | Quadratic | 0.8420 | .. |  |
|  | Cubic | 0.1734 | .. |  |
|  | General health |  |  |  |
|  | Linear | 0.3232 | .. |  |
|  | Quadratic | 0.1884 | .. |  |
|  | Cubic | 0.0374 | .. |  |
|  | Quartic | 0.7321 | .. |  |
|  |  |  |  |  |

Tests were done using orthogonal polynomial contrasts.

# ***Table S6:* Risk factors for breakthrough SARS-CoV-2 infection in the pre-booster cohort, stratified by primary vaccination course and COVID-19 test frequency**

|  |  | **Stratified by primary vaccination course** | | **Stratified by COVID-19 test frequency** | |  |
| --- | --- | --- | --- | --- | --- | --- |
|  |  | HR (95% CI) | p value | HR (95% CI) | p value |  |
|  | Age, years | 0.97 (0.96–0.97) | <0.0001 | 0.97 (0.96–0.97) | <0.0001 |  |
|  | Sex |  |  |  |  |  |
|  | Female | 1.00 |  | 1.00 |  |  |
|  | Male | 1.05 (0.91–1.21) | 0.5383 | 1.05 (0.91–1.22) | 0.4866 |  |
|  | Highest educational level attained |  |  |  |  |  |
|  | Post-grad | 1.00 |  | 1.00 |  |  |
|  | College or university | 1.23 (1.00–1.50) | 0.0463 | 1.13 (0.98–1.31) | 0.0966 |  |
|  | Higher or further (A levels) | 1.23 (1.00–1.50) | 0.0463 | 1.22 (1.00–1.50) | 0.0505 |  |
|  | Primary or secondary | 1.79 (1.45–2.21) | <0.0001 | 1.77 (1.43–2.19) | <0.0001 |  |
|  | Frontline worker |  |  |  |  |  |
|  | No | 1.00 |  | 1.00 |  |  |
|  | Non-health | 1.13 (0.95–1.35) | 0.1644 | 1.13 (0.95–1.35) | 0.1619 |  |
|  | Health | 0.64 (0.50–0.83) | 0.0006 | 0.64 (0.50–0.82) | 0.0005 |  |
|  | Housing |  |  |  |  |  |
|  | Owns own home | 1.00 |  | 1.00 |  |  |
|  | Mortgage | 1.09 (0.92–1.29) | 0.3085 | 1.09 (0.92–1.29) | 0.2988 |  |
|  | Privately renting | 0.83 (0.63–1.10) | 0.1898 | 0.82 (0.62–1.09) | 0.1713 |  |
|  | Renting from council | 1.34 (0.96–1.89) | 0.0895 | 1.34 (0.95–1.88) | 0.0934 |  |
|  | Other | 0.89 (0.63–1.25) | 0.4919 | 0.86 (0.61–1.20) | 0.3766 |  |
|  | Number of people per bedroom |  |  |  |  |  |
|  | <1 | 1.00 |  | 1.00 |  |  |
|  | 1 to <2 | 1.16 (0.99–1.34) | 0.0619 | 1.15 (0.99–1.34) | 0.0621 |  |
|  | ≥2 | 1.71 (1.23–2.38) | 0.0016 | 1.70 (1.22–2.37) | 0.0016 |  |
|  | Multigenerational households |  |  |  |  |  |
|  | Living alone | 0.88 (0.70–1.11) | 0.2737 | 0.89 (0.71–1.11) | 0.2969 |  |
|  | Single generation | 1.00 |  | 1.00 |  |  |
|  | Two or more generations | 1.04 (0.86–1.25) | 0.6884 | 1.04 (0.86–1.26) | 0.6625 |  |
|  | Shares home with schoolchildren (5–15 years) |  |  |  |  |  |
|  | No | 1.00 |  | 1.00 |  |  |
|  | Yes | 1.36 (1.08–1.71) | 0.0082 | 1.38 (1.10–1.73) | 0.0057 |  |
|  | Shares home with working-age adult (16–64 year) |  |  |  |  |  |
|  | No | 1.00 |  | 1.00 |  |  |
|  | Yes | 1.00 (0.83–1.20) | 0.9635 | 1.00 (0.83–1.20) | 0.9787 |  |
|  | Alcohol consumption, units per week |  |  |  |  |  |
|  | 0 | 1.00 |  | 1.00 |  |  |
|  | 1–7 | 1.05 (0.90–1.23) | 0.5569 | 1.05 (0.90–1.23) | 0.5509 |  |
|  | 8–14 | 1.15 (0.96–1.38) | 0.1402 | 1.16 (0.97–1.39) | 0.1064 |  |
|  | ≥15 | 1.17 (0.96–1.43) | 0.1192 | 1.17 (0.96–1.43) | 0.1125 |  |
|  | Occasional or daily vaping |  |  |  |  |  |
|  | No | 1.00 |  | 1.00 |  |  |
|  | Yes | 1.60 (1.21–2.11) | 0.0010 | 1.60 (1.21–2.11) | 0.0009 |  |
|  | Any visits to or from other households in past week |  |  |  |  |  |
|  | No | 1.00 |  | 1.00 |  |  |
|  | Yes | 1.19 (1.02–1.40) | 0.0318 | 1.19 (1.01–1.40) | 0.0343 |  |
|  | Weekly visits to shops |  |  |  |  |  |
|  | 0 | 1.00 |  | 1.00 |  |  |
|  | 1 | 1.01 (0.73–1.42) | 0.9351 | 1.02 (0.73–1.42) | 0.9141 |  |
|  | 2–3 | 1.15 (0.85–1.55) | 0.3766 | 1.14 (0.84–1.54) | 0.3984 |  |
|  | ≥4 | 1.10 (0.81–1.50) | 0.5400 | 1.09 (0.81–1.49) | 0.5625 |  |
|  | Weekly visits to other indoor public places (not shops) |  |  |  |  |  |
|  | 0 | 1.00 |  | 1.00 |  |  |
|  | 1–2 | 1.17 (0.98–1.39) | 0.0782 | 1.17 (0.99–1.40) | 0.0701 |  |
|  | ≥3 | 1.35 (1.13–1.63) | 0.0013 | 1.36 (1.13–1.63) | 0.0012 |  |
|  | Weekly SARS-CoV-2 incidence (per 1000 people) | 1.04 (1.03–1.06) | <0.0001 | 1.04 (1.03–1.06) | <0.0001 |  |
|  | Inter-vaccine interval, weeks | 1.09 (1.07–1.11) | <0.0001 | 1.08 (1.06–1.09) | <0.0001 |  |
|  | Primary vaccination course |  |  |  |  |  |
|  | ChAdOx1 | .. | .. | 1.64 (1.42–1.90) | <0.0001 |  |
|  | BNT162b2 | .. |  | 1.00 |  |  |
|  | Season of first vaccination |  |  |  |  |  |
|  | Mid-October to mid-April (Winter) | 1.00 |  | 1.00 |  |  |
|  | Mid-April to mid-October (Summer) | 2.07 (1.65–2.59) | <0.0001 | 2.01 (1.61–2.52) | <0.0001 |  |
|  | Previous infection |  |  |  |  |  |
|  | No | 1.00 |  | 1.00 |  |  |
|  | Yes | 0.54 (0.39–0.75) | 0.0002 | 0.54 (0.39–0.74) | 0.0001 |  |
|  | Daily portions of fruit, vegetables, and salad |  |  |  |  |  |
|  | 0–2 | 1.00 |  | 1.00 |  |  |
|  | 3–4 | 1.15 (0.94–1.40) | 0.1750 | 1.15 (0.94–1.40) | 0.1713 |  |
|  | 5 | 1.07 (0.86–1.34) | 0.5342 | 1.08 (0.86–1.35) | 0.4984 |  |
|  | ≥6 | 1.20 (0.98–1.47) | 0.0792 | 1.21 (0.99–1.48) | 0.0660 |  |
|  | General health |  |  |  |  |  |
|  | Excellent | 1.00 |  | 1.00 |  |  |
|  | Very good | 0.86 (0.73–1.02) | 0.0875 | 0.85 (0.72–1.01) | 0.0630 |  |
|  | Good | 0.95 (0.79–1.15) | 0.6041 | 0.95 (0.79–1.14) | 0.5536 |  |
|  | Fair | 1.11 (0.88–1.40) | 0.3732 | 1.11 (0.88–1.40) | 0.3844 |  |
|  | Poor | 0.93 (0.64–1.36) | 0.7262 | 0.92 (0.63–1.35) | 0.6773 |  |
|  | Hypertension |  |  |  |  |  |
|  | No | 1.00 |  | 1.00 |  |  |
|  | Yes | 0.97 (0.80–1.19) | 0.7963 | 0.98 (0.80–1.20) | 0.8476 |  |
|  | Immunodeficiency |  |  |  |  |  |
|  | No | 1.00 |  | 1.00 |  |  |
|  | Yes | 0.15 (0.02–1.10) | 0.0623 | 0.15 (0.02–1.10) | 0.0628 |  |
|  | Calcium channel blockers |  |  |  |  |  |
|  | No | 1.00 |  | 1.00 |  |  |
|  | Yes | 0.85 (0.64–1.15) | 0.2927 | 0.85 (0.63–1.14) | 0.2708 |  |
|  | Anticholinergics |  |  |  |  |  |
|  | No | 1.00 |  | 1.00 |  |  |
|  | Yes | 0.73 (0.52–1.01) | 0.0600 | 0.73 (0.53–1.02) | 0.0649 |  |
|  | Reported COVID-19 test in every questionnaire |  |  |  |  |  |
|  | No | 1.00 |  | .. |  |  |
|  | Yes | 2.04 (1.77–2.35) | <0.0001 | .. | .. |  |
|  |  |  |  |  |  |  |

Stratified analyses were done in 14,273 participants, with 1022 breakthrough infections. ChAdOx1 = ChAdOx1 nCoV-19. HR = hazard ratio.

# ***Table S7:* Risk factors for breakthrough infection in the post-booster cohort, stratified by probiotics and angiotensin receptor blockers**

|  |  | **Stratified by probiotics** | | **Stratified by angiotensin receptor blockers** | |  |
| --- | --- | --- | --- | --- | --- | --- |
|  |  | HR (95% CI) | p value | HR (95% CI) | p value |  |
|  | Age, years | 0.97 (0.97–0.98) | <0.0001 | 0.97 (0.97–0.98) | <0.0001 |  |
|  | Sex |  |  |  |  |  |
|  | Female | 1.00 |  | 1.00 |  |  |
|  | Male | 0.86 (0.74–1.00) | 0.0543 | 0.86 (0.74–1.00) | 0.0495 |  |
|  | Ethnicity |  |  |  |  |  |
|  | White | 1.00 |  | 1.00 |  |  |
|  | Mixed, multiple, or other ethnic groups | 0.28 (0.14–0.57) | 0.0004 | 0.28 (0.14–0.57) | 0.0004 |  |
|  | South Asian | 0.46 (0.22–0.97) | 0.0427 | 0.46 (0.22–0.97) | 0.0424 |  |
|  | Black, African, Caribbean, or Black British | 0.95 (0.35–2.56) | 0.9211 | 0.95 (0.35–2.55) | 0.9135 |  |
|  | Highest educational level attained |  |  |  |  |  |
|  | Post-graduate | 1.00 |  | 1.00 |  |  |
|  | College or university | 1.09 (0.94–1.26) | 0.2542 | 1.09 (0.94–1.26) | 0.2537 |  |
|  | Higher or further (A levels) | 1.30 (1.06–1.58) | 0.0112 | 1.30 (1.06–1.58) | 0.0112 |  |
|  | Primary or secondary | 1.46 (1.16–1.83) | 0.0014 | 1.46 (1.16–1.83) | 0.0014 |  |
|  | Frontline worker |  |  |  |  |  |
|  | No | 1.00 |  | 1.00 |  |  |
|  | Non-health | 1.05 (0.86–1.30) | 0.6208 | 1.05 (0.85–1.30) | 0.6310 |  |
|  | Health or care | 0.91 (0.71–1.16) | 0.4413 | 0.91 (0.71–1.16) | 0.4403 |  |
|  | Housing |  |  |  |  |  |
|  | Owns own home | 1.00 |  | 1.00 |  |  |
|  | Mortgage | 1.21 (1.02–1.44) | 0.0253 | 1.22 (1.03–1.44) | 0.0237 |  |
|  | Privately renting | 1.47 (1.11–1.95) | 0.0072 | 1.47 (1.11–1.95) | 0.0071 |  |
|  | Renting from council | 1.13 (0.72–1.78) | 0.5877 | 1.14 (0.73–1.79) | 0.5677 |  |
|  | Other | 1.14 (0.75–1.75) | 0.5413 | 1.14 (0.75–1.75) | 0.5398 |  |
|  | Multigenerational households |  |  |  |  |  |
|  | Living alone | 0.90 (0.73–1.12) | 0.3536 | 0.91 (0.73–1.12) | 0.3592 |  |
|  | Single generation | 1.00 |  | 1.00 |  |  |
|  | Two or more generations | 0.98 (0.82–1.17) | 0.7987 | 0.98 (0.82–1.17) | 0.8002 |  |
|  | Shares home with schoolchildren (5–15 years) |  |  |  |  |  |
|  | No | 1.00 |  | 1.00 |  |  |
|  | Yes | 1.36 (1.06–1.74) | 0.0165 | 1.35 (1.05–1.73) | 0.0178 |  |
|  | Shares home with working-age adults (16–64 years) |  |  |  |  |  |
|  | No | 1.00 |  | 1.00 |  |  |
|  | Yes | 1.26 (1.06–1.50) | 0.0080 | 1.27 (1.07–1.51) | 0.0074 |  |
|  | Alcohol consumption, units per week |  |  |  |  |  |
|  | 0 | 1.00 |  | 1.00 |  |  |
|  | 1–7 | 1.04 (0.88–1.23) | 0.6671 | 1.04 (0.88–1.23) | 0.6529 |  |
|  | 8–14 | 0.98 (0.81–1.19) | 0.8165 | 0.98 (0.81–1.19) | 0.8334 |  |
|  | ≥15 | 1.26 (1.04–1.53) | 0.0187 | 1.27 (1.04–1.54) | 0.0161 |  |
|  | Travel outside of the UK since last questionnaire |  |  |  |  |  |
|  | No | 1.00 |  | 1.00 |  |  |
|  | Yes | 1.07 (0.92–1.26) | 0.3780 | 1.07 (0.92–1.26) | 0.3816 |  |
|  | Weekly journeys on public transport |  |  |  |  |  |
|  | 0 | 1.00 |  | 1.00 |  |  |
|  | 1–5 | 1.00 (0.85–1.17) | 0.9624 | 0.99 (0.85–1.17) | 0.9404 |  |
|  | ≥6 | 1.16 (0.92–1.46) | 0.2238 | 1.15 (0.91–1.46) | 0.2357 |  |
|  | Any visits to or from other households in past week |  |  |  |  |  |
|  | No | 1.00 |  | 1.00 |  |  |
|  | Yes | 1.07 (0.91–1.27) | 0.4132 | 1.07 (0.91–1.26) | 0.4323 |  |
|  | Weekly visits to shops |  |  |  |  |  |
|  | 0 | 1.00 |  | 1.00 |  |  |
|  | 1 | 0.91 (0.65–1.27) | 0.5718 | 0.91 (0.65–1.27) | 0.5861 |  |
|  | 2–3 | 1.04 (0.77–1.40) | 0.7924 | 1.05 (0.78–1.41) | 0.7545 |  |
|  | ≥4 | 1.17 (0.87–1.58) | 0.2926 | 1.18 (0.88–1.59) | 0.2723 |  |
|  | Weekly visits to other indoor public places (not shops) |  |  |  |  |  |
|  | None | 1.00 |  | 1.00 |  |  |
|  | 1–2 | 1.09 (0.91–1.30) | 0.3693 | 1.08 (0.91–1.30) | 0.3739 |  |
|  | ≥3 | 1.29 (1.07–1.56) | 0.0078 | 1.30 (1.07–1.57) | 0.0070 |  |
|  | Vigorous physical exercise, h per week |  |  |  |  |  |
|  | 0 | 0.89 (0.75–1.06) | 0.1911 | 0.89 (0.75–1.06) | 0.1899 |  |
|  | 1 | 0.89 (0.76–1.05) | 0.1650 | 0.89 (0.76–1.05) | 0.1596 |  |
|  | 2 | 1.00 |  | 1.00 |  |  |
|  | Actual sleep, h/night |  |  |  |  |  |
|  | ≤5 | 0.75 (0.59–0.95) | 0.0195 | 0.75 (0.59–0.96) | 0.0202 |  |
|  | 6 | 1.01 (0.87–1.17) | 0.9090 | 1.01 (0.87–1.18) | 0.8689 |  |
|  | 7 | 1.00 |  | 1.00 |  |  |
|  | ≥8 | 1.03 (0.87–1.22) | 0.7200 | 1.03 (0.87–1.22) | 0.7167 |  |
|  | Weekly SARS-CoV-2 incidence | 1.09 (1.08–1.10) | <0.0001 | 1.09 (1.08–1.10) | <0.0001 |  |
|  | Inter-vaccine interval (primary to booster), weeks | 0.98 (0.96–1.01) | 0.2029 | 0.98 (0.96–1.01) | 0.2079 |  |
|  | Combination of primary and booster vaccinations |  |  |  |  |  |
|  | ChAdOx1 plus BNT162b2 booster | 1.06 (0.91–1.23) | 0.4774 | 1.06 (0.91–1.23) | 0.4847 |  |
|  | ChAdOx1 plus mRNA-1273 booster | 1.26 (1.00–1.57) | 0.0465 | 1.25 (1.00–1.56) | 0.0522 |  |
|  | BNT162b2 plus BNT162b2 booster | 1.00 |  | 1.00 |  |  |
|  | BNT162b2 plus mRNA-1273 booster | 0.99 (0.67–1.45) | 0.9418 | 0.98 (0.67–1.44) | 0.9204 |  |
|  | Season of booster vaccination |  |  |  |  |  |
|  | Mid-October to mid-April (Winter) | 1.00 |  | 1.00 |  |  |
|  | Mid-April to mid-October (Summer) | 0.56 (0.46–0.67) | <0.0001 | 0.56 (0.46–0.68) | <0.0001 |  |
|  | Previous infection |  |  |  |  |  |
|  | No evidence of previous infection | 1.00 |  | 1.00 |  |  |
|  | Previous infection before primary course of vaccination | 0.75 (0.55–1.01) | 0.0598 | 0.74 (0.55–1.01) | 0.0565 |  |
|  | Previous infection after primary course of vaccination | 0.28 (0.16–0.47) | <0.0001 | 0.28 (0.16–0.47) | <0.0001 |  |
|  | Probiotics |  |  |  |  |  |
|  | No | .. |  | 1.00 |  |  |
|  | Yes | .. | .. | 0.79 (0.57–1.09) | 0.1480 |  |
|  | Fish oil, krill oil, or other omega-3 supplements |  |  |  |  |  |
|  | No | 1.00 |  | 1.00 |  |  |
|  | Yes | 0.80 (0.64–1.00) | 0.0531 | 0.80 (0.64–1.00) | 0.0494 |  |
|  | Cod liver oil supplements |  |  |  |  |  |
|  | No | 1.00 |  | 1.00 |  |  |
|  | Yes | 0.80 (0.61–1.04) | 0.0985 | 0.79 (0.61–1.04) | 0.0934 |  |
|  | Atopy |  |  |  |  |  |
|  | No | 1.00 |  | 1.00 |  |  |
|  | Yes | 0.89 (0.77–1.03) | 0.1212 | 0.89 (0.77–1.03) | 0.1103 |  |
|  | Diabetes types |  |  |  |  |  |
|  | No diabetes | 1.00 |  | 1.00 |  |  |
|  | Pre-diabetes | 0.84 (0.55–1.29) | 0.4257 | 0.84 (0.55–1.28) | 0.4125 |  |
|  | Type 1 diabetes | 0.88 (0.44–1.78) | 0.7270 | 0.88 (0.44–1.77) | 0.7189 |  |
|  | Type 2 diabetes | 0.89 (0.52–1.54) | 0.6807 | 0.89 (0.52–1.53) | 0.6712 |  |
|  | Beta blockers |  |  |  |  |  |
|  | No | 1.00 |  | 1.00 |  |  |
|  | Yes | 0.86 (0.65–1.15) | 0.3134 | 0.87 (0.65–1.15) | 0.3214 |  |
|  | ACE inhibitors |  |  |  |  |  |
|  | No | 1.00 |  | 1.00 |  |  |
|  | Yes | 0.98 (0.77–1.25) | 0.8771 | 0.98 (0.77–1.25) | 0.8830 |  |
|  | Angiotensin receptor blockers |  |  |  |  |  |
|  | No | 1.00 |  | .. |  |  |
|  | Yes | 0.93 (0.69–1.27) | 0.6622 | .. | .. |  |
|  | Thiazides |  |  |  |  |  |
|  | No | 1.00 |  | 1.00 |  |  |
|  | Yes | 0.62 (0.37–1.03) | 0.0625 | 0.62 (0.38–1.03) | 0.0635 |  |
|  | Metformin |  |  |  |  |  |
|  | No | 1.00 |  | 1.00 |  |  |
|  | Yes | 0.87 (0.44–1.72) | 0.6819 | 0.86 (0.43–1.70) | 0.6580 |  |
|  | Reported COVID-19 test in every questionnaire |  |  |  |  |  |
|  | No | 1.00 |  | 1.00 |  |  |
|  | Yes | 1.97 (1.74–2.24) | <0.0001 | 1.97 (1.74–2.24) | <0.0001 |  |
|  |  |  |  |  |  |  |

Stratified analyses were done in 10,590 participants, with 997 breakthrough infections. ACE = angiotensin-converting-enzyme. ChAdOx1 = ChAdOx1 nCoV-19. HR = hazard ratio.

# ***Table S8:* Risk factors for breakthrough SARS-CoV-2 infection in the post-primary cohort, omitting schoolchildren variable**

Likelihood ratio test comparing model without schoolchildren variable with full model:
p <0.0057

|  |  | **HR (95% CI)** | **p value** |  |
| --- | --- | --- | --- | --- |
|  | Age, years | 0.96 (0.96–0.97) | <0.0001 |  |
|  | Sex |  |  |  |
|  | Female | 1.00 |  |  |
|  | Male | 1.04 (0.90–1.20) | 0.5890 |  |
|  | Highest educational level attained |  |  |  |
|  | Post-graduate | 1.00 |  |  |
|  | College or university | 1.14 (0.99–1.33) | 0.0769 |  |
|  | Higher or further (A levels) | 1.23 (1.01–1.51) | 0.0422 |  |
|  | Primary or secondary | 1.80 (1.46–2.23) | <0.0001 |  |
|  | Frontline worker |  |  |  |
|  | No | 1.00 |  |  |
|  | Non-health | 1.15 (0.96–1.37) | 0.1295 |  |
|  | Health | 0.66 (0.52–0.85) | 0.0012 |  |
|  | Housing |  |  |  |
|  | Owns own home | 1.00 |  |  |
|  | Mortgage | 1.13 (0.96–1.34) | 0.1361 |  |
|  | Privately renting | 0.82 (0.62–1.08) | 0.1628 |  |
|  | Renting from council | 1.35 (0.96–1.89) | 0.0874 |  |
|  | Other | 0.85 (0.61–1.19) | 0.3353 |  |
|  | Number of people per bedroom |  |  |  |
|  | <1 | 1.00 |  |  |
|  | 1 to <2 | 1.19 (1.03–1.38) | 0.0217 |  |
|  | ≥2 | 1.76 (1.27–2.45) | 0.0008 |  |
|  | Multigenerational households |  |  |  |
|  | Living alone | 0.90 (0.71–1.13) | 0.3502 |  |
|  | Single generation | 1.00 |  |  |
|  | Two or more generations | 1.22 (1.06–1.41) | 0.0049 |  |
|  | Shares home with working-age adult (16–64 year) |  |  |  |
|  | No | 1.00 |  |  |
|  | Yes | 1.00 (0.83–1.20) | 0.9952 |  |
|  | Alcohol consumption, units per week |  |  |  |
|  | 0 | 1.00 |  |  |
|  | 1–7 | 1.05 (0.90–1.23) | 0.5258 |  |
|  | 8–14 | 1.15 (0.96–1.38) | 0.1377 |  |
|  | ≥15 | 1.17 (0.96–1.43) | 0.1185 |  |
|  | Occasional or daily vaping |  |  |  |
|  | No | 1.00 |  |  |
|  | Yes | 1.60 (1.21–2.12) | 0.0009 |  |
|  | Any visits to or from other households in past week |  |  |  |
|  | No | 1.00 |  |  |
|  | Yes | 1.19 (1.02–1.40) | 0.0319 |  |
|  | Weekly visits to shops |  |  |  |
|  | 0 | 1.00 |  |  |
|  | 1 | 1.02 (0.73–1.42) | 0.9233 |  |
|  | 2–3 | 1.15 (0.85–1.56) | 0.3684 |  |
|  | ≥4 | 1.11 (0.81–1.50) | 0.5165 |  |
|  | Weekly visits to other indoor public places (not shops) |  |  |  |
|  | 0 | 1.00 |  |  |
|  | 1–2 | 1.17 (0.98–1.39) | 0.0756 |  |
|  | ≥3 | 1.35 (1.13–1.63) | 0.0013 |  |
|  | Weekly SARS-CoV-2 incidence (per 1000 people) | 1.05 (1.03–1.06) | <0.0001 |  |
|  | Inter-vaccine interval, weeks | 1.08 (1.06–1.10) | <0.0001 |  |
|  | Primary vaccination course |  |  |  |
|  | ChAdOx1 | 1.64 (1.42–1.89) | <0.0001 |  |
|  | BNT162b2 | 1.00 |  |  |
|  | Season of first vaccination |  |  |  |
|  | Mid-October to mid-April (Winter) | 1.00 |  |  |
|  | Mid-April to mid-October (Summer) | 2.00 (1.59–2.51) | <0.0001 |  |
|  | Previous infection |  |  |  |
|  | No | 1.00 |  |  |
|  | Yes | 0.54 (0.39–0.74) | 0.0002 |  |
|  | Daily portions of fruit, vegetables, and salad |  |  |  |
|  | 0–2 | 1.00 |  |  |
|  | 3–4 | 1.16 (0.95–1.42) | 0.1371 |  |
|  | 5 | 1.08 (0.87–1.35) | 0.4788 |  |
|  | ≥6 | 1.21 (0.99–1.48) | 0.0632 |  |
|  | General health |  |  |  |
|  | Excellent | 1.00 |  |  |
|  | Very good | 0.86 (0.72–1.02) | 0.0739 |  |
|  | Good | 0.95 (0.79–1.14) | 0.5708 |  |
|  | Fair | 1.11 (0.88–1.40) | 0.3804 |  |
|  | Poor | 0.95 (0.65–1.38) | 0.7738 |  |
|  | Hypertension |  |  |  |
|  | No | 1.00 |  |  |
|  | Yes | 0.97 (0.79–1.19) | 0.7885 |  |
|  | Immunodeficiency |  |  |  |
|  | No | 1.00 |  |  |
|  | Yes | 0.15 (0.02–1.10) | 0.0624 |  |
|  | Calcium channel blockers |  |  |  |
|  | No | 1.00 |  |  |
|  | Yes | 0.85 (0.63–1.14) | 0.2875 |  |
|  | Anticholinergics |  |  |  |
|  | No | 1.00 |  |  |
|  | Yes | 0.73 (0.52–1.01) | 0.0567 |  |
|  | Reported COVID-19 test in every questionnaire |  |  |  |
|  | No | 1.00 |  |  |
|  | Yes | 2.05 (1.78–2.36) | <0.0001 |  |
|  |  |  |  |  |

The analysis was done in 14,273 participants, with 1022 breakthrough infections. ChAdOx1 = ChAdOx1 nCoV-19. HR = hazard ratio.

# ***Table S9:* Risk factors for breakthrough SARS-CoV-2 infection in the post-booster cohort, omitting schoolchildren and metformin variables**

Likelihood ratio test comparing model without schoolchildren or metformin variables with full model:
p=0.0538

|  |  | **Minimally adjusted** |  |  |
| --- | --- | --- | --- | --- |
|  |  | HR (95% CI) | p value |  |
|  | Age, years | 0.97 (0.96–0.98) | <0.0001 |  |
|  | Sex |  |  |  |
|  | Female | 1.00 |  |  |
|  | Male | 0.86 (0.74–1.00) | 0.0530 |  |
|  | Ethnicity |  |  |  |
|  | White | 1.00 |  |  |
|  | Mixed, multiple, or other ethnic groups | 0.28 (0.14–0.57) | 0.0004 |  |
|  | South Asian | 0.46 (0.22–0.97) | 0.0424 |  |
|  | Black, African, Caribbean, or Black British | 0.98 (0.36–2.62) | 0.9612 |  |
|  | Highest educational level attained |  |  |  |
|  | Post-graduate | 1.00 |  |  |
|  | College or university | 1.09 (0.94–1.26) | 0.2641 |  |
|  | Higher or further (A levels) | 1.29 (1.06–1.58) | 0.0116 |  |
|  | Primary or secondary | 1.46 (1.16–1.83) | 0.0014 |  |
|  | Frontline worker |  |  |  |
|  | No | 1.00 |  |  |
|  | Non-health | 1.07 (0.87–1.31) | 0.5466 |  |
|  | Health or care | 0.91 (0.71–1.16) | 0.4508 |  |
|  | Housing |  |  |  |
|  | Owns own home | 1.00 |  |  |
|  | Mortgage | 1.25 (1.06–1.48) | 0.0087 |  |
|  | Privately renting | 1.45 (1.09–1.92) | 0.0101 |  |
|  | Renting from council | 1.16 (0.74–1.81) | 0.5228 |  |
|  | Other | 1.11 (0.72–1.69) | 0.6452 |  |
|  | Multigenerational households |  |  |  |
|  | Living alone | 0.91 (0.73–1.12) | 0.3653 |  |
|  | Single generation | 1.00 |  |  |
|  | Two or more generations | 1.10 (0.95–1.27) | 0.2096 |  |
|  | Shares home with working-age adults (16–64 years) |  |  |  |
|  | No | 1.00 |  |  |
|  | Yes | 1.25 (1.06–1.49) | 0.0100 |  |
|  | Alcohol consumption, units per week |  |  |  |
|  | 0 | 1.00 |  |  |
|  | 1–7 | 1.04 (0.88–1.23) | 0.6728 |  |
|  | 8–14 | 0.98 (0.81–1.19) | 0.8324 |  |
|  | ≥15 | 1.25 (1.03–1.52) | 0.0216 |  |
|  | Travelled outside of the UK since last questionnaire |  |  |  |
|  | No | 1.00 |  |  |
|  | Yes | 1.06 (0.91–1.25) | 0.4421 |  |
|  | Weekly journeys on public transport |  |  |  |
|  | 0 | 1.00 |  |  |
|  | 1–5 | 0.99 (0.85–1.17) | 0.9315 |  |
|  | ≥6 | 1.14 (0.90–1.44) | 0.2659 |  |
|  | Any visits to or from other households in past week |  |  |  |
|  | No | 1.00 |  |  |
|  | Yes | 1.08 (0.91–1.27) | 0.3921 |  |
|  | Weekly visits to shops |  |  |  |
|  | 0 | 1.00 |  |  |
|  | 1 | 0.91 (0.65–1.27) | 0.5897 |  |
|  | 2–3 | 1.05 (0.78–1.41) | 0.7487 |  |
|  | ≥4 | 1.19 (0.89–1.60) | 0.2475 |  |
|  | Weekly visits to other indoor public places (not shops) |  |  |  |
|  | 0 | 1.00 |  |  |
|  | 1–2 | 1.08 (0.91–1.30) | 0.3742 |  |
|  | ≥3 | 1.29 (1.07–1.56) | 0.0080 |  |
|  | Vigorous physical exercise, h per week |  |  |  |
|  | 0 | 0.89 (0.75–1.06) | 0.1943 |  |
|  | 1 | 0.90 (0.77–1.05) | 0.1697 |  |
|  | 2 | 1.00 |  |  |
|  | Actual sleep, h per night |  |  |  |
|  | ≤5 | 0.75 (0.59–0.96) | 0.0201 |  |
|  | 6 | 1.01 (0.87–1.18) | 0.8482 |  |
|  | 7 | 1.00 |  |  |
|  | ≥8 | 1.03 (0.87–1.22) | 0.7444 |  |
|  | Weekly SARS-CoV-2 incidence | 1.09 (1.08–1.10) | <0.0001 |  |
|  | Inter-vaccine interval (primary to booster), weeks | 0.99 (0.96–1.01) | 0.2172 |  |
|  | Combination of primary and booster vaccinations |  |  |  |
|  | ChAdOx1 plus BNT162b2 booster | 1.06 (0.91–1.24) | 0.4182 |  |
|  | ChAdOx1 plus mRNA-1273 booster | 1.26 (1.00–1.57) | 0.0456 |  |
|  | BNT162b2 plus BNT162b2 booster | 1.00 |  |  |
|  | BNT162b2 plus mRNA-1273 booster | 0.99 (0.67–1.46) | 0.9702 |  |
|  | Season of booster vaccination |  |  |  |
|  | Mid-October to mid-April (Winter) | 1.00 |  |  |
|  | Mid-April to mid-October (Summer) | 0.56 (0.46–0.68) | <0.0001 |  |
|  | Previous infection |  |  |  |
|  | No evidence of previous infection | 1.00 |  |  |
|  | Previous infection before primary course of vaccination | 0.75 (0.55–1.02) | 0.0664 |  |
|  | Previous infection after primary course of vaccination | 0.28 (0.16–0.47) | <0.0001 |  |
|  | Probiotics |  |  |  |
|  | No | 1.00 |  |  |
|  | Yes | 0.79 (0.57–1.08) | 0.1438 |  |
|  | Fish oil, krill oil, or other omega-3 supplements |  |  |  |
|  | No | 1.00 |  |  |
|  | Yes | 0.80 (0.63–1.00) | 0.0468 |  |
|  | Cod liver oil supplements |  |  |  |
|  | No | 1.00 |  |  |
|  | Yes | 0.80 (0.61–1.05) | 0.1018 |  |
|  | Atopy |  |  |  |
|  | No | 1.00 |  |  |
|  | Yes | 0.89 (0.77–1.03) | 0.1073 |  |
|  | Diabetes |  |  |  |
|  | No diabetes | 1.00 |  |  |
|  | Pre-diabetes | 0.84 (0.55–1.29) | 0.4339 |  |
|  | Type 1 diabetes | 0.85 (0.42–1.71) | 0.6519 |  |
|  | Type 2 diabetes | 0.82 (0.56–1.20) | 0.3041 |  |
|  | Beta blockers |  |  |  |
|  | No | 1.00 |  |  |
|  | Yes | 0.86 (0.65–1.14) | 0.2881 |  |
|  | ACE inhibitors |  |  |  |
|  | No | 1.00 |  |  |
|  | Yes | 0.97 (0.76–1.24) | 0.8269 |  |
|  | Angiotensin receptor blockers |  |  |  |
|  | No | 1.00 |  |  |
|  | Yes | 0.93 (0.69–1.26) | 0.6484 |  |
|  | Thiazides |  |  |  |
|  | No | 1.00 |  |  |
|  | Yes | 0.62 (0.38–1.03) | 0.0636 |  |
|  | Reported COVID-19 test in every questionnaire |  |  |  |
|  | No | 1.00 |  |  |
|  | Yes | 1.98 (1.74–2.25) | <0.0001 |  |
|  |  |  |  |  |

The analysis was done in 10,590 participants, with 997 breakthrough infections. ACE = angiotensin-converting-enzyme. ChAdOx1 = ChAdOx1 nCoV-19. HR = hazard ratio.

# ***Table S10:* Sensitivity analysis: Risk factors for breakthrough SARS-CoV-2 infection in the post-primary cohort, censored at date of booster dose**

|  |  | **HR (95% CI)** | **p value** |  |
| --- | --- | --- | --- | --- |
|  | Age, years | 0.97 (0.96–0.97) | <0.0001 |  |
|  | Sex |  |  |  |
|  | Female | 1.00 |  |  |
|  | Male | 1.03 (0.88–1.20) | 0.7330 |  |
|  | Highest educational level attained |  |  |  |
|  | Post-graduate | 1.00 |  |  |
|  | College or university | 1.19 (1.02–1.38) | 0.0308 |  |
|  | Higher or further (A levels) | 1.17 (0.95–1.45) | 0.1447 |  |
|  | Primary or secondary | 1.73 (1.38–2.17) | <0.0001 |  |
|  | Frontline worker |  |  |  |
|  | No | 1.00 |  |  |
|  | Non-health | 1.10 (0.92–1.33) | 0.2937 |  |
|  | Health | 0.61 (0.47–0.80) | 0.0002 |  |
|  | Housing |  |  |  |
|  | Owns own home | 1.00 |  |  |
|  | Mortgage | 1.13 (0.95–1.35) | 0.1652 |  |
|  | Privately renting | 0.85 (0.64–1.14) | 0.2787 |  |
|  | Renting from council | 1.43 (1.01–2.04) | 0.0462 |  |
|  | Other | 0.92 (0.65–1.31) | 0.6536 |  |
|  | Number of people per bedroom |  |  |  |
|  | <1 | 1.00 |  |  |
|  | 1 to <2 | 1.12 (0.96–1.32) | 0.1529 |  |
|  | ≥2 | 1.66 (1.18–2.36) | 0.0041 |  |
|  | Multigenerational households |  |  |  |
|  | Living alone | 0.88 (0.69–1.12) | 0.3064 |  |
|  | Single generation | 1.00 |  |  |
|  | Two or more generations | 1.07 (0.88–1.29) | 0.5260 |  |
|  | Shares home with schoolchildren (5–15 years) |  |  |  |
|  | No | 1.00 |  |  |
|  | Yes | 1.40 (1.10–1.77) | 0.0056 |  |
|  | Shares home with working-age adult (16–64 year) |  |  |  |
|  | No | 1.00 |  |  |
|  | Yes | 1.02 (0.84–1.24) | 0.8143 |  |
|  | Quartiles of IMD rank |  |  |  |
|  | Q4 (least deprived) | 1.00 |  |  |
|  | Q3 | 0.88 (0.74–1.06) | 0.1948 |  |
|  | Q2 | 1.00 (0.83–1.19) | 0.9681 |  |
|  | Q1 (most deprived) | 0.97 (0.80–1.17) | 0.7531 |  |
|  | Alcohol consumption, units per week |  |  |  |
|  | 0 | 1.00 |  |  |
|  | 1–7 | 1.06 (0.90–1.25) | 0.5042 |  |
|  | 8–14 | 1.13 (0.94–1.37) | 0.1972 |  |
|  | ≥15 | 1.19 (0.97–1.46) | 0.1044 |  |
|  | Occasional or daily vaping |  |  |  |
|  | No | 1.00 |  |  |
|  | Yes | 1.62 (1.21–2.16) | 0.0013 |  |
|  | Any visits to or from other households in past week |  |  |  |
|  | No | 1.00 |  |  |
|  | Yes | 1.18 (1.00–1.40) | 0.0496 |  |
|  | Weekly visits to shops |  |  |  |
|  | 0 | 1.00 |  |  |
|  | 1 | 0.98 (0.69–1.38) | 0.9049 |  |
|  | 2–3 | 1.10 (0.81–1.51) | 0.5399 |  |
|  | ≥4 | 1.07 (0.78–1.47) | 0.6740 |  |
|  | Weekly visits to other indoor public places (not shops) |  |  |  |
|  | 0 | 1.00 |  |  |
|  | 1–2 | 1.16 (0.97–1.40) | 0.1032 |  |
|  | ≥3 | 1.37 (1.13–1.66) | 0.0014 |  |
|  | Weekly SARS-CoV-2 incidence (per 1000 people) | 1.04 (1.03–1.06) | <0.0001 |  |
|  | Inter-vaccine interval, weeks | 1.08 (1.06–1.10) | <0.0001 |  |
|  | Primary vaccination course |  |  |  |
|  | ChAdOx1 | 1.65 (1.41–1.92) | <0.0001 |  |
|  | BNT162b2 | 1.00 |  |  |
|  | Season of first vaccination |  |  |  |
|  | Mid-October to mid-April (Winter) | 1.00 |  |  |
|  | Mid-April to mid-October (Summer) | 1.86 (1.47–2.36) | <0.0001 |  |
|  | Previous infection |  |  |  |
|  | No | 1.00 |  |  |
|  | Yes | 0.55 (0.39–0.76) | 0.0004 |  |
|  | Daily portions of fruit, vegetables, and salad |  |  |  |
|  | 0–2 | 1.00 |  |  |
|  | 3–4 | 1.16 (0.94–1.44) | 0.1578 |  |
|  | 5 | 1.10 (0.87–1.39) | 0.4208 |  |
|  | ≥6 | 1.23 (0.99–1.52) | 0.0595 |  |
|  | General health |  |  |  |
|  | Excellent | 1.00 |  |  |
|  | Very good | 0.88 (0.74–1.06) | 0.1719 |  |
|  | Good | 0.93 (0.77–1.13) | 0.4819 |  |
|  | Fair | 1.14 (0.90–1.46) | 0.2753 |  |
|  | Poor | 0.79 (0.52–1.19) | 0.2587 |  |
|  | Hypertension |  |  |  |
|  | No | 1.00 |  |  |
|  | Yes | 0.94 (0.76–1.17) | 0.5781 |  |
|  | Immunodeficiency |  |  |  |
|  | No | 1.00 |  |  |
|  | Yes | 0.17 (0.02–1.18) | 0.0732 |  |
|  | Calcium channel blockers |  |  |  |
|  | No | 1.00 |  |  |
|  | Yes | 0.88 (0.65–1.20) | 0.4183 |  |
|  | Anticholinergics |  |  |  |
|  | No | 1.00 |  |  |
|  | Yes | 0.76 (0.54–1.07) | 0.1152 |  |
|  | Reported COVID-19 test in every questionnaire |  |  |  |
|  | No | 1.00 |  |  |
|  | Yes | 2.09 (1.80–2.43) | <0.0001 |  |
|  |  |  |  |  |

The analysis was done in 14,222 participants, with 931 breakthrough infections. ChAdOx1 = ChAdOx1 nCoV-19. HR = hazard ratio.

# ***Table S11:* Sensitivity analysis: Risk factors for breakthrough SARS-CoV-2 infection in the post-primary cohort (pre-Omicron)**

|  |  | **Fully adjusted model** | |  |
| --- | --- | --- | --- | --- |
|  |  | HR (95% CI) | p value |  |
|  | Age (years) | 0.97 (0.96–0.98) | <0.0001 |  |
|  | Sex |  |  |  |
|  | Female | 1.00 |  |  |
|  | Male | 1.09 (0.92–1.30) | 0.2947 |  |
|  | Highest educational level attained |  |  |  |
|  | Post-graduate | 1.00 |  |  |
|  | College or university | 1.13 (0.95–1.34) | 0.1757 |  |
|  | Higher or further (A levels) | 1.07 (0.84–1.36) | 0.6054 |  |
|  | Primary or secondary | 1.77 (1.38–2.26) | <0.0001 |  |
|  | Frontline worker |  |  |  |
|  | No | 1.00 |  |  |
|  | Non-health | 1.17 (0.95–1.44) | 0.1379 |  |
|  | Health or care | 0.63 (0.46–0.86) | 0.0041 |  |
|  | Housing |  |  |  |
|  | Owns own home | 1.00 |  |  |
|  | Mortgage | 1.09 (0.90–1.33) | 0.3785 |  |
|  | Privately renting | 0.97 (0.70–1.35) | 0.8575 |  |
|  | Renting from council | 1.29 (0.85–1.97) | 0.2302 |  |
|  | Other | 0.98 (0.64–1.49) | 0.9072 |  |
|  | Number of people per bedroom |  |  |  |
|  | <1 | 1.00 |  |  |
|  | 1 to <2 | 1.09 (0.91–1.31) | 0.3488 |  |
|  | ≥2 | 2.02 (1.39–2.96) | 0.0003 |  |
|  | Multigenerational households |  |  |  |
|  | Living alone | 0.85 (0.66–1.11) | 0.2298 |  |
|  | Single generation | 1.00 |  |  |
|  | Two or more generations | 1.02 (0.82–1.26) | 0.8822 |  |
|  | Shares home with schoolchildren (5–15 years) |  |  |  |
|  | No | 1.00 |  |  |
|  | Yes | 1.64 (1.25–2.15) | 0.0003 |  |
|  | Shares home with working-age adults (16–64 years) |  |  |  |
|  | No | 1.00 |  |  |
|  | Yes | 0.97 (0.79–1.19) | 0.7580 |  |
|  | Alcohol consumption, units per week |  |  |  |
|  | 0 | 1.00 |  |  |
|  | 1–7 | 1.12 (0.93–1.35) | 0.2294 |  |
|  | 8–14 | 1.19 (0.96–1.47) | 0.1185 |  |
|  | ≥15 | 1.20 (0.96–1.51) | 0.1167 |  |
|  | Occasional or daily vaping |  |  |  |
|  | No | 1.00 |  |  |
|  | Yes | 1.73 (1.26–2.39) | 0.0008 |  |
|  | Any visits to or from other households in past week |  |  |  |
|  | No | 1.00 |  |  |
|  | Yes | 1.28 (1.06–1.55) | 0.0106 |  |
|  | Weekly visits to other indoor public places (not shops) |  |  |  |
|  | 0 | 1.00 |  |  |
|  | 1–2 | 1.17 (0.99–1.40) | 0.0731 |  |
|  | ≥3 | 1.36 (1.13–1.63) | 0.0012 |  |
|  | Actual sleep, h per night |  |  |  |
|  | ≤5 | 1.04 (0.81–1.33) | 0.7586 |  |
|  | 6 | 1.02 (0.85–1.22) | 0.8475 |  |
|  | 7 | 1.00 |  |  |
|  | ≥8 | 0.86 (0.71–1.04) | 0.1109 |  |
|  | Weekly SARS-CoV-2 incidence (per 1000 people) | 0.96 (0.93–0.99) | 0.0028 |  |
|  | Inter-vaccine interval, weeks | 1.06 (1.02–1.10) | 0.0008 |  |
|  | Primary vaccination course |  |  |  |
|  | ChAdOx1 | 1.91 (1.60–2.27) | <0.0001 |  |
|  | BNT162b2 | 1.00 |  |  |
|  | Season of first vaccination |  |  |  |
|  | Mid-October to mid-April (Winter) | 1.00 |  |  |
|  | Mid-April to mid-October (Summer) | 1.97 (1.46–2.65) | <0.0001 |  |
|  | Previous infection |  |  |  |
|  | No | 1.00 |  |  |
|  | Yes | 0.57 (0.38–0.85) | 0.0059 |  |
|  | Daily portions of fruit, vegetables, and salad |  |  |  |
|  | 0–2 | 1.00 |  |  |
|  | 3–4 | 1.20 (0.94–1.53) | 0.1486 |  |
|  | 5 | 1.12 (0.86–1.47) | 0.4069 |  |
|  | ≥6 | 1.27 (0.99–1.62) | 0.0560 |  |
|  | General health |  |  |  |
|  | Excellent | 1.00 |  |  |
|  | Very good | 0.92 (0.75–1.12) | 0.4168 |  |
|  | Good | 0.89 (0.71–1.12) | 0.3167 |  |
|  | Fair | 0.96 (0.72–1.27) | 0.7560 |  |
|  | Poor | 0.74 (0.45–1.21) | 0.2324 |  |
|  | Hypertension |  |  |  |
|  | No | 1.00 |  |  |
|  | Yes | 0.98 (0.76–1.27) | 0.8854 |  |
|  | ACE inhibitors |  |  |  |
|  | No | 1.00 |  |  |
|  | Yes | 0.91 (0.65–1.27) | 0.5748 |  |
|  | Calcium channel blockers |  |  |  |
|  | No | 1.00 |  |  |
|  | Yes | 0.80 (0.57–1.13) | 0.2101 |  |
|  | Sodium-glucose co-transporter-2 inhibitors |  |  |  |
|  | No | 1.00 |  |  |
|  | Yes | 2.86 (1.34–6.09) | 0.0065 |  |
|  | Anticholinergics |  |  |  |
|  | No | 1.00 |  |  |
|  | Yes | 0.68 (0.46–1.02) | 0.0613 |  |
|  | Reported COVID-19 test in every questionnaire |  |  |  |
|  | No | 1.00 |  |  |
|  | Yes | 2.18 (1.85–2.57) | <0.0001 |  |
|  |  |  |  |  |

The analysis was done in 14,254 participants, with 745 breakthrough infections. Participants were censored at date of booster vaccination, breakthrough infection, or Dec 15, 2021 (approximate date of Omicron dominance in the UK), whichever came first. ACE = angiotensin-converting-enzyme. ChAdOx1 = ChAdOx1 nCoV-19. HR = hazard ratio.

# ***Table S12:* Sensitivity analysis: Risk factors for breakthrough SARS-CoV-2 infection in the post-booster cohort (post-Omicron)**

|  |  | **Fully adjusted model** | |  |
| --- | --- | --- | --- | --- |
|  |  | HR (95% CI) | p value |  |
|  | Age, years | 0.98 (0.97–0.99) | <0.0001 |  |
|  | Sex |  |  |  |
|  | Female | 1.00 |  |  |
|  | Male | 0.84 (0.72–0.98) | 0.0301 |  |
|  | Ethnicity |  |  |  |
|  | White | 1.00 |  |  |
|  | Mixed, multiple, or other ethnic groups | 0.30 (0.15–0.60) | 0.0007 |  |
|  | South Asian | 0.49 (0.23–1.04) | 0.0625 |  |
|  | Black, African, Caribbean, or Black British | 0.93 (0.34–2.49) | 0.8800 |  |
|  | Housing |  |  |  |
|  | Owns own home | 1.00 |  |  |
|  | Mortgage | 1.19 (1.00–1.41) | 0.0481 |  |
|  | Privately renting | 1.46 (1.09–1.96) | 0.0116 |  |
|  | Renting from council | 1.25 (0.79–1.97) | 0.3492 |  |
|  | Other | 1.08 (0.69–1.68) | 0.7287 |  |
|  | Multigenerational households |  |  |  |
|  | Living alone | 0.88 (0.71–1.10) | 0.2726 |  |
|  | Single generation | 1.00 |  |  |
|  | Two or more generations | 0.98 (0.82–1.18) | 0.8430 |  |
|  | Shares home with schoolchildren (5–15 years) |  |  |  |
|  | No | 1.00 |  |  |
|  | Yes | 1.34 (1.04–1.73) | 0.0233 |  |
|  | Shares home with working-age adults (16–64 years) |  |  |  |
|  | No | 1.00 |  |  |
|  | Yes | 1.22 (1.02–1.45) | 0.0310 |  |
|  | Alcohol consumption, units per week |  |  |  |
|  | 0 | 1.00 |  |  |
|  | 1–7 | 1.03 (0.87–1.23) | 0.7111 |  |
|  | 8–14 | 0.97 (0.80–1.19) | 0.8033 |  |
|  | ≥15 | 1.28 (1.05–1.56) | 0.0150 |  |
|  | Travelled outside of the UK since last questionnaire |  |  |  |
|  | No | 1.00 |  |  |
|  | Yes | 1.11 (0.95–1.31) | 0.2010 |  |
|  | Weekly journeys on public transport |  |  |  |
|  | 0 | 1.00 |  |  |
|  | 1–5 | 1.04 (0.88–1.22) | 0.6706 |  |
|  | ≥6 | 1.18 (0.92–1.51) | 0.1961 |  |
|  | Any visits to or from other households in past week |  |  |  |
|  | No | 1.00 |  |  |
|  | Yes | 1.11 (0.93–1.32) | 0.2383 |  |
|  | Weekly visits to shops |  |  |  |
|  | 0 | 1.00 |  |  |
|  | 1 | 0.93 (0.66–1.32) | 0.6995 |  |
|  | 2–3 | 1.07 (0.79–1.45) | 0.6627 |  |
|  | ≥4 | 1.26 (0.93–1.71) | 0.1380 |  |
|  | Weekly visits to other indoor public places (not shops) |  |  |  |
|  | 0 | 1.00 |  |  |
|  | 1–2 | 1.04 (0.87–1.25) | 0.6511 |  |
|  | ≥3 | 1.24 (1.02–1.50) | 0.0319 |  |
|  | Vigorous physical exercise, h per week |  |  |  |
|  | 0 | 0.86 (0.72–1.03) | 0.1096 |  |
|  | 1 | 0.88 (0.75–1.04) | 0.1302 |  |
|  | 2 | 1.00 |  |  |
|  | Actual sleep, h per night |  |  |  |
|  | ≤5 | 0.82 (0.64–1.05) | 0.1107 |  |
|  | 6 | 1.04 (0.89–1.22) | 0.5875 |  |
|  | 7 | 1.00 |  |  |
|  | ≥8 | 1.06 (0.89–1.27) | 0.4891 |  |
|  | Weekly SARS-CoV-2 incidence | 1.10 (1.09–1.11) | <0.0001 |  |
|  | Weeks since booster vaccination | 1.06 (1.02–1.10) | 0.0017 |  |
|  | Combination of primary and booster vaccinations |  |  |  |
|  | ChAdOx1 plus BNT162b2 booster | 0.96 (0.82–1.12) | 0.6000 |  |
|  | ChAdOx1 plus mRNA-1273 booster | 0.95 (0.75–1.21) | 0.6765 |  |
|  | BNT162b2 plus BNT162b2 booster | 1.00 |  |  |
|  | BNT162b2 plus mRNA-1273 booster | 0.78 (0.52–1.16) | 0.2163 |  |
|  | Season of booster vaccination |  |  |  |
|  | Mid-October to mid-April (Winter) | 1.00 |  |  |
|  | Mid-April to mid-October (Summer) | 0.80 (0.62–1.04) | 0.1014 |  |
|  | Previous infection |  |  |  |
|  | No evidence of previous infection | 1.00 |  |  |
|  | Previous infection before primary course of vaccination | 0.74 (0.54–1.01) | 0.0586 |  |
|  | Previous infection after primary course of vaccination | 0.26 (0.15–0.45) | <0.0001 |  |
|  | Probiotics |  |  |  |
|  | No | 1.00 |  |  |
|  | Yes | 0.81 (0.59–1.12) | 0.2067 |  |
|  | Fish oil, krill oil, or other omega-3 supplements |  |  |  |
|  | No | 1.00 |  |  |
|  | Yes | 0.82 (0.65–1.04) | 0.1017 |  |
|  | Cod liver oil supplements |  |  |  |
|  | No | 1.00 |  |  |
|  | Yes | 0.80 (0.60–1.05) | 0.1086 |  |
|  | General health |  |  |  |
|  | Excellent | 1.00 |  |  |
|  | Very good | 1.03 (0.85–1.26) | 0.7344 |  |
|  | Good | 1.04 (0.84–1.29) | 0.6937 |  |
|  | Fair | 0.99 (0.76–1.30) | 0.9586 |  |
|  | Poor | 1.04 (0.68–1.59) | 0.8388 |  |
|  | Atopy |  |  |  |
|  | No | 1.00 |  |  |
|  | Yes | 0.89 (0.76–1.03) | 0.1183 |  |
|  | Thiazides |  |  |  |
|  | No | 1.00 |  |  |
|  | Yes | 0.58 (0.35–0.97) | 0.0372 |  |
|  | Metformin |  |  |  |
|  | No | 1.00 |  |  |
|  | Yes | 0.85 (0.53–1.36) | 0.5008 |  |
|  | Reported COVID-19 test in every questionnaire |  |  |  |
|  | No | 1.00 |  |  |
|  | Yes | 1.92 (1.68–2.19) | <0.0001 |  |
|  |  |  |  |  |

The analysis was done in 10,420 participants, with 939 breakthrough infections. Participants were included in the analysis at 14 days after their booster dose or from Dec 15, 2021 (approximate date of Omicron dominance in the UK), whichever came later. Analyses were adjusted for time since booster dose (at time of entry into the analysis). ChAdOx1 = ChAdOx1 nCoV-19. HR = hazard ratio.

# ***Table S13:* Exploratory analysis: Risk factors for breakthrough SARS-CoV-2 infection in the post-primary cohort, including interactions for age and weekly SARS-CoV-2 incidence**

Likelihood ratio test comparing full model with interactions model:
p=0.0004

|  |  | **HR (95% CI)** | **p value** |  |
| --- | --- | --- | --- | --- |
|  | **Main effects** |  |  |  |
|  | Age, years | 0.97 (0.96–0.97) | <0.0001 |  |
|  | Sex |  |  |  |
|  | Female | 1.00 |  |  |
|  | Male | 1.05 (0.90–1.21) | 0.5461 |  |
|  | Highest educational level attained |  |  |  |
|  | Post-graduate | 1.00 |  |  |
|  | College or university | 1.14 (0.98–1.32) | 0.0831 |  |
|  | Higher or further (A levels) | 1.23 (1.00–1.50) | 0.0461 |  |
|  | Primary or secondary | 1.78 (1.44–2.20) | <0.0001 |  |
|  | Frontline worker |  |  |  |
|  | No | 1.00 |  |  |
|  | Non-health | 1.13 (0.95–1.35) | 0.1709 |  |
|  | Health or care | 0.66 (0.52–0.85) | 0.0013 |  |
|  | Housing |  |  |  |
|  | Owns own home | 1.00 |  |  |
|  | Mortgage | 1.09 (0.92–1.29) | 0.2977 |  |
|  | Privately renting | 0.83 (0.63–1.10) | 0.1962 |  |
|  | Renting from council | 1.32 (0.94–1.86) | 0.1119 |  |
|  | Other | 0.86 (0.61–1.21) | 0.3862 |  |
|  | Number of people per bedroom |  |  |  |
|  | <1 | 1.00 |  |  |
|  | 1 to <2 | 1.11 (0.93–1.33) | 0.2414 |  |
|  | ≥2 | 2.36 (1.54–3.60) | <0.0001 |  |
|  | Multigenerational households |  |  |  |
|  | Living alone | 0.88 (0.70–1.10) | 0.2610 |  |
|  | Single generation | 1.00 |  |  |
|  | Two or more generations | 1.04 (0.86–1.25) | 0.7116 |  |
|  | Shares home with schoolchildren (5–15 years) |  |  |  |
|  | No | 1.00 |  |  |
|  | Yes | 1.74 (1.30–2.34) | 0.0002 |  |
|  | Shares home with working-age adults (16–64 years) |  |  |  |
|  | No | 1.00 |  |  |
|  | Yes | 1.00 (0.83–1.21) | 0.9807 |  |
|  | Alcohol consumption, units per week |  |  |  |
|  | 0 | 1.00 |  |  |
|  | 1–7 | 1.04 (0.89–1.22) | 0.6120 |  |
|  | 8–14 | 1.14 (0.95–1.36) | 0.1686 |  |
|  | ≥15 | 1.16 (0.95–1.41) | 0.1440 |  |
|  | Occasional or daily vaping |  |  |  |
|  | No | 1.00 |  |  |
|  | Yes | 1.58 (1.20–2.09) | 0.0012 |  |
|  | Any visits to or from other households in past week |  |  |  |
|  | No | 1.00 |  |  |
|  | Yes | 1.19 (1.01–1.39) | 0.0359 |  |
|  | Weekly visits to shops |  |  |  |
|  | 0 | 1.00 |  |  |
|  | 1 | 1.02 (0.73–1.42) | 0.9072 |  |
|  | 2–3 | 1.15 (0.85–1.55) | 0.3750 |  |
|  | ≥4 | 1.10 (0.81–1.49) | 0.5504 |  |
|  | Weekly visits to other indoor public places (not shops) |  |  |  |
|  | 0 | 1.00 |  |  |
|  | 1–2 | 1.17 (0.98–1.39) | 0.0830 |  |
|  | ≥3 | 1.35 (1.12–1.62) | 0.0015 |  |
|  | Weekly SARS-CoV-2 incidence (per 1000 people) | 1.08 (1.06–1.10) | <0.0001 |  |
|  | Inter-vaccine interval, weeks | 1.08 (1.06–1.09) | <0.0001 |  |
|  | Primary vaccination course |  |  |  |
|  | ChAdOx1 | 2.10 (1.70–2.61) | <0.0001 |  |
|  | BNT162b2 | 1.00 |  |  |
|  | Season of first vaccination |  |  |  |
|  | Mid-October to mid-April (Winter) | 1.00 |  |  |
|  | Mid-April to mid-October (Summer) | 1.97 (1.56–2.47) | <0.0001 |  |
|  | Previous infection |  |  |  |
|  | No | 1.00 |  |  |
|  | Yes | 0.54 (0.40–0.75) | 0.0002 |  |
|  | Daily portions of fruit, vegetables, and salad |  |  |  |
|  | 0–2 | 1.00 |  |  |
|  | 3–4 | 1.15 (0.95–1.41) | 0.1583 |  |
|  | 5 | 1.07 (0.86–1.34) | 0.5292 |  |
|  | ≥6 | 1.20 (0.98–1.47) | 0.0804 |  |
|  | General health |  |  |  |
|  | Excellent | 1.00 |  |  |
|  | Very good | 0.87 (0.74–1.03) | 0.1173 |  |
|  | Good | 0.96 (0.79–1.15) | 0.6415 |  |
|  | Fair | 1.12 (0.89–1.41) | 0.3459 |  |
|  | Poor | 0.94 (0.64–1.37) | 0.7485 |  |
|  | Hypertension |  |  |  |
|  | No | 1.00 |  |  |
|  | Yes | 0.97 (0.79–1.19) | 0.7647 |  |
|  | Immunodeficiency |  |  |  |
|  | No | 1.00 |  |  |
|  | Yes | 0.15 (0.02–1.10) | 0.0619 |  |
|  | Calcium channel blockers |  |  |  |
|  | No | 1.00 |  |  |
|  | Yes | 0.85 (0.63–1.14) | 0.2850 |  |
|  | Anticholinergics |  |  |  |
|  | No | 1.00 |  |  |
|  | Yes | 0.73 (0.52–1.01) | 0.0587 |  |
|  | Reported COVID-19 test in every questionnaire |  |  |  |
|  | No | 1.00 |  |  |
|  | Yes | 2.02 (1.75–2.33) | <0.0001 |  |
|  | **Interactions** |  |  |  |
|  | Number of people per bedroom ∗ Age (years) |  |  |  |
|  | <1 | 1.00 |  |  |
|  | 1 to <2 | 1.00 (0.99–1.01) | 0.7006 |  |
|  | ≥2 | 1.02 (1.00–1.04) | 0.0356 |  |
|  | Primary vaccination course ∗ Weekly SARS-CoV-2 incidence (per 1000 people) |  |  |  |
|  | ChAdOx1 | 0.96 (0.94–0.99) | 0.0011 |  |
|  | BNT162b2 | 1.00 |  |  |
|  | Shares home with schoolchildren (5–15 years) ∗ Weekly SARS-CoV-2 incidence (per 1000 people) |  |  |  |
|  | No | 1.00 |  |  |
|  | Yes | 0.97 (0.94–0.99) | 0.0174 |  |
|  |  |  |  |  |

The analysis was done in 14,273 participants, with 1022 breakthrough infections. Age is mean centred to aid with interpretation. ChAdOx1 = ChAdOx1 nCoV-19. HR = hazard ratio.

# ***Table S14:* Exploratory analysis: Risk factors for breakthrough SARS-CoV-2 infection in the post-booster cohort, including interactions for age and weekly SARS-CoV-2 incidence**

Likelihood ratio test comparing full model with interactions model:
p=0.0047

|  |  | **HR (95% CI)** | **p value** |  |
| --- | --- | --- | --- | --- |
|  | **Main effects** |  |  |  |
|  | Age, years | 0.96 (0.95–0.98) | <0.0001 |  |
|  | Sex |  |  |  |
|  | Female | 1.00 |  |  |
|  | Male | 0.86 (0.74–1.00) | 0.0498 |  |
|  | Ethnicity |  |  |  |
|  | White | 1.00 |  |  |
|  | Mixed, multiple, or other ethnic groups | 0.29 (0.14–0.58) | 0.0005 |  |
|  | South Asian | 0.45 (0.21–0.95) | 0.0373 |  |
|  | Black, African, Caribbean, or Black British | 0.98 (0.36–2.63) | 0.9633 |  |
|  | Highest educational level attained |  |  |  |
|  | Post-grad uate | 1.00 |  |  |
|  | College or university | 1.09 (0.94–1.26) | 0.2620 |  |
|  | Higher or further (A levels) | 1.31 (1.07–1.60) | 0.0086 |  |
|  | Primary or secondary | 1.46 (1.16–1.84) | 0.0013 |  |
|  | Frontline worker |  |  |  |
|  | No | 1.00 |  |  |
|  | Non-health | 1.07 (0.86–1.31) | 0.5489 |  |
|  | Health or care | 0.93 (0.73–1.20) | 0.5816 |  |
|  | Housing |  |  |  |
|  | Owns own home | 1.00 |  |  |
|  | Mortgage | 1.20 (1.01–1.42) | 0.0353 |  |
|  | Privately renting | 1.47 (1.11–1.94) | 0.0077 |  |
|  | Renting from council | 1.11 (0.71–1.74) | 0.6503 |  |
|  | Other | 1.16 (0.76–1.77) | 0.5012 |  |
|  | Multigenerational households |  |  |  |
|  | Living alone | 0.91 (0.74–1.12) | 0.3747 |  |
|  | Single generation | 1.00 |  |  |
|  | Two or more generations | 0.98 (0.82–1.17) | 0.8061 |  |
|  | Shares home with schoolchildren (5–15 years) |  |  |  |
|  | No | 1.00 |  |  |
|  | Yes | 1.33 (1.04–1.71) | 0.0249 |  |
|  | Shares home with working-age adults (16–64 years) |  |  |  |
|  | No | 1.00 |  |  |
|  | Yes | 1.28 (1.08–1.52) | 0.0054 |  |
|  | Alcohol consumption, units per week |  |  |  |
|  | 0 | 1.00 |  |  |
|  | 1–7 | 1.05 (0.88–1.24) | 0.5936 |  |
|  | 8–14 | 0.99 (0.81–1.20) | 0.8911 |  |
|  | ≥15 | 1.27 (1.05–1.54) | 0.0154 |  |
|  | Travelled outside of the UK since last questionnaire |  |  |  |
|  | No | 1.00 |  |  |
|  | Yes | 1.08 (0.92–1.27) | 0.3353 |  |
|  | Weekly journeys on public transport |  |  |  |
|  | 0 | 1.00 |  |  |
|  | 1–5 | 0.99 (0.85–1.16) | 0.9270 |  |
|  | ≥6 | 1.17 (0.92–1.47) | 0.1980 |  |
|  | Any visits to or from other households in past week |  |  |  |
|  | No | 1.00 |  |  |
|  | Yes | 1.08 (0.91–1.28) | 0.3638 |  |
|  | Weekly visits to shops |  |  |  |
|  | 0 | 1.00 |  |  |
|  | 1 | 0.90 (0.64–1.25) | 0.5261 |  |
|  | 2–3 | 1.03 (0.77–1.39) | 0.8222 |  |
|  | ≥4 | 1.17 (0.87–1.57) | 0.3114 |  |
|  | Weekly visits to other indoor public places (not shops) |  |  |  |
|  | 0 | 1.00 |  |  |
|  | 1–2 | 1.20 (0.98–1.46) | 0.0781 |  |
|  | ≥3 | 1.46 (1.18–1.80) | 0.0004 |  |
|  | Vigorous physical exercise, h per week |  |  |  |
|  | 0 | 0.88 (0.74–1.05) | 0.1674 |  |
|  | 1 | 0.90 (0.77–1.05) | 0.1695 |  |
|  | 2 | 1.00 |  |  |
|  | Actual sleep, h per night |  |  |  |
|  | ≤5 | 0.75 (0.59–0.95) | 0.0178 |  |
|  | 6 | 1.01 (0.87–1.17) | 0.9013 |  |
|  | 7 | 1.00 |  |  |
|  | ≥8 | 1.04 (0.87–1.23) | 0.6827 |  |
|  | Weekly SARS-CoV-2 incidence | 1.09 (1.08–1.10) | <0.0001 |  |
|  | Weeks since booster vaccination | 0.98 (0.96–1.01) | 0.1331 |  |
|  | Combination of primary and booster vaccinations |  |  |  |
|  | ChAdOx1 plus BNT162b2 booster | 1.05 (0.90–1.22) | 0.5519 |  |
|  | ChAdOx1 plus mRNA-1273 booster | 1.05 (0.79–1.38) | 0.7468 |  |
|  | BNT162b2 plus BNT162b2 booster | 1.00 |  |  |
|  | BNT162b2 plus mRNA-1273 booster | 1.15 (0.74–1.78) | 0.5330 |  |
|  | Season of booster vaccination |  |  |  |
|  | Mid-October to mid-April (Winter) | 1.00 |  |  |
|  | Mid-April to mid-October (Summer) | 0.56 (0.46–0.67) | <0.0001 |  |
|  | Previous infection |  |  |  |
|  | No evidence of previous infection | 1.00 |  |  |
|  | Previous infection before primary course of vaccination | 0.75 (0.56–1.02) | 0.0691 |  |
|  | Previous infection after primary course of vaccination | 0.27 (0.16–0.46) | <0.0001 |  |
|  | Probiotics |  |  |  |
|  | No | 1.00 |  |  |
|  | Yes | 0.78 (0.56–1.07) | 0.1251 |  |
|  | Fish oil, krill oil, or other omega-3 supplements |  |  |  |
|  | No | 1.00 |  |  |
|  | Yes | 0.79 (0.63–1.00) | 0.0462 |  |
|  | Cod liver oil supplements |  |  |  |
|  | No | 1.00 |  |  |
|  | Yes | 0.79 (0.60–1.03) | 0.0809 |  |
|  | Atopy |  |  |  |
|  | No | 1.00 |  |  |
|  | Yes | 0.89 (0.77–1.03) | 0.1195 |  |
|  | Diabetes |  |  |  |
|  | No diabetes | 1.00 |  |  |
|  | Pre-diabetes | 0.83 (0.54–1.28) | 0.4044 |  |
|  | Type 1 diabetes | 0.88 (0.44–1.78) | 0.7215 |  |
|  | Type 2 diabetes | 0.89 (0.52–1.54) | 0.6898 |  |
|  | Beta blockers |  |  |  |
|  | No | 1.00 |  |  |
|  | Yes | 0.87 (0.65–1.15) | 0.3321 |  |
|  | ACE inhibitors |  |  |  |
|  | No | 1.00 |  |  |
|  | Yes | 0.98 (0.77–1.25) | 0.8572 |  |
|  | Angiotensin receptor blockers |  |  |  |
|  | No | 1.00 |  |  |
|  | Yes | 0.94 (0.69–1.27) | 0.6852 |  |
|  | Thiazides |  |  |  |
|  | No | 1.00 |  |  |
|  | Yes | 0.63 (0.38–1.04) | 0.0686 |  |
|  | Metformin |  |  |  |
|  | No | 1.00 |  |  |
|  | Yes | 0.86 (0.43–1.70) | 0.6659 |  |
|  | Reported COVID-19 test in every questionnaire |  |  |  |
|  | No | 1.00 |  |  |
|  | Yes | 1.97 (1.73–2.24) | <0.0001 |  |
|  | **Interactions** |  |  |  |
|  | Weekly visits to other indoor public places ∗ Age, years |  |  |  |
|  | 0 | 1.00 |  |  |
|  | 1–2 | 1.02 (1.00–1.03) | 0.0212 |  |
|  | ≥3 | 1.02 (1.01–1.04) | 0.0027 |  |
|  | Combination of primary and booster vaccinations ∗ Age, years |  |  |  |
|  | ChAdOx1 plus BNT162b2 booster | 1.00 (0.99–1.01) | 0.6242 |  |
|  | ChAdOx1 plus mRNA-1273 booster | 0.98 (0.96–1.00) | 0.0135 |  |
|  | BNT162b2 plus BNT162b2 booster | 1.00 |  |  |
|  | BNT162b2 plus mRNA-1273 booster | 1.01 (0.99–1.04) | 0.3424 |  |
|  |  |  |  |  |

The analysis was done in 10,590 participants, with 997 breakthrough infections. Age is mean centred to aid with interpretation. ACE = angiotensin-converting-enzyme. ChAdOx1 = ChAdOx1 nCoV-19. HR = hazard ratio.

# ***Table S15:* Missing data**

|  |  | **Post-primary analysis** | **Post-booster analysis** |  |
| --- | --- | --- | --- | --- |
|  | **Baseline** | | |  |
|  | Ethnicity | <0.1% | <0.1% |  |
|  | Highest educational level attained | 0.1% | 0.1% |  |
|  | Frontline worker | 0.1% | <0.1% |  |
|  | Housing status | <0.1% | <0.1% |  |
|  | Number of people per bedroom | 0.7% | 0.7% |  |
|  | Multigenerational households | 0.4% | 0.3% |  |
|  | Schoolchildren (5–15 years) at home with participant | 0.2% | 0.2% |  |
|  | Working-age adult (16–64 years) at home with participant | 0.2% | 0.2% |  |
|  | Quartiles of IMD rank | 0.4% | 0.1% |  |
|  | Claiming Universal Credit | 0.3% | 0.3% |  |
|  | Dog at home | 0.1% | 0.1% |  |
|  | Environmental tobacco smoke | <0.1% | <0.1% |  |
|  | Daily portions of fruit, vegetables, and salad | 0.2% | 0.3% |  |
|  | Daily portions of dairy products or calcium-fortified alternatives | 0.3% | 0.3% |  |
|  | BMI, kg/m2 | 0.2% | 0.1% |  |
|  | Diabetes type | <0.1% | 0.1% |  |
|  | Periodontitis | 8.8% | 4.2% |  |
|  | BCG vaccination | 9.6% | 9.4% |  |
|  | **Peri-vaccination** | | |  |
|  | General health | 6.8% | <0.1% |  |
|  | Actual sleep | 6.8% | 0.2% |  |
|  | Lower impact physical exercise | 7.0% | 0.4% |  |
|  | Light physical exercise | 6.9% | 0.2% |  |
|  | Vigorous physical exercise | 6.9% | 0.4% |  |
|  | Alcohol consumption | 6.7% | <0.1% |  |
|  | Smoking status | 6.7% | <0.1% |  |
|  | Vaping status | 6.8% | <0.1% |  |
|  | Multivitamin supplements | 6.8% | 0.1% |  |
|  | Vitamin A supplements | 6.8% | 0.1% |  |
|  | Vitamin C supplements | 6.8% | 0.1% |  |
|  | Vitamin D supplements | 6.8% | 0.1% |  |
|  | Cod liver oil supplements | 6.8% | 0.1% |  |
|  | Fish oil, krill oil, or other omega-3 supplements | 6.8% | 0.1% |  |
|  | Garlic or allicin supplements | 6.8% | 0.1% |  |
|  | Iron supplements | 6.8% | 0.1% |  |
|  | Probiotics | 6.8% | 0.1% |  |
|  | Selenium supplements | 6.8% | 0.1% |  |
|  | Zinc supplements | 6.8% | 0.1% |  |
|  | **Time varying** | | |  |
|  | Any visits to or from other households in past week | 0.1% | <0.1% |  |
|  | Weekly visits to shops | 0.2% | <0.1% |  |
|  | Weekly visits to other indoor public places (not shops) | 0.2% | 0.1% |  |
|  | Weekly journeys on public transport | 0.1% | <0.1% |  |
|  |  |  |  |  |

Only variables with missing data are shown. A greater number of participants in the post-primary analysis were missing peri-vaccination data, as they joined the cohort after receiving their first vaccination. Participants with missing baseline data were excluded from any minimally adjusted or fully adjusted analyses including those variables. Missing data for peri-vaccination variables were replaced with baseline values. Missing data for time-varying factors were replaced with average values over the follow-up period, if available; if unavailable, missing data were replaced with values from the last available observation before the start of the follow-up period, or baseline values. IMD = Index of Multiple Deprivation. BMI = body-mass index. BCG = Bacille Calmette Guérin.

# ***Table S16:* Monthly response rates**

|  |  | **All questionnaires completed** | **10% missing** | **20% missing** | **30% missing** | **>30% missing** |  |
| --- | --- | --- | --- | --- | --- | --- | --- |
|  | Post-primary analysis (n=14,713) | 12,320 (83.7%) | 123 (0.8%) | 370 (2.5%) | 499 (3.4%) | 1401 (9.5%) |  |
|  | Post-booster analysis (n=10,665) | 9970 (93.5%) | 0 | 8 (<0.1%) | 159 (1.5%) | 528 (5.0%) |  |
|  |  |  |  |  |  |  |  |

Percentage of missing questionnaires is calculated over all monthly questionnaires sent out during each participant's follow-up period. Follow-up periods for participants reached a maximum of 13 questionnaires in the post-primary analysis and six questionnaires in the post-booster analysis. The wording of the questions ("Since you last checked in with us…"; see table S2) encourages participants to report any tests, vaccinations, and other key events that have taken place since they submitted their last questionnaire, with no time limit.
